# A single-molecule view of transcription reveals convoys of RNA polymerases and multi-scale bursting

**DOI:** 10.1038/ncomms12248

**Published:** 2016-07-27

**Authors:** Katjana Tantale, Florian Mueller, Alja Kozulic-Pirher, Annick Lesne, Jean-Marc Victor, Marie-Cécile Robert, Serena Capozi, Racha Chouaib, Volker Bäcker, Julio Mateos-Langerak, Xavier Darzacq, Christophe Zimmer, Eugenia Basyuk, Edouard Bertrand

**Affiliations:** 1 Institut de Génétique Moléculaire de Montpellier, CNRS UMR5535, 1919, route de Mende, 34293 Montpellier Cedex 5, France; 2 Unité Imagerie et Modélisation, Département Biologie Cellulaire et Infections, Institut Pasteur and CNRS UMR 3691, 28, rue du Docteur Roux, 75015 Paris, France; 3 Laboratoire de Physique Théorique de la Matière Condensée; CNRS UMR 7600, UPMC-Paris 6, Sorbonne Universités, 4 place Jussieu, 75252 Paris Cedex 5, France; 4 BioCampus Montpellier, CNRS UMS3426, 141, rue de la Cardonille, 34094 Montpellier Cedex 5, France; 5 Ecole Normale Supérieure, CNRS UMR 8197 Paris, France

## Abstract

Live-cell imaging has revealed unexpected features of gene expression. Here using improved single-molecule RNA microscopy, we show that synthesis of HIV-1 RNA is achieved by groups of closely spaced polymerases, termed convoys, as opposed to single isolated enzymes. Convoys arise by a Mediator-dependent reinitiation mechanism, which generates a transient but rapid succession of polymerases initiating and escaping the promoter. During elongation, polymerases are spaced by few hundred nucleotides, and physical modelling suggests that DNA torsional stress may maintain polymerase spacing. We additionally observe that the HIV-1 promoter displays stochastic fluctuations on two time scales, which we refer to as multi-scale bursting. Each time scale is regulated independently: Mediator controls minute-scale fluctuation (convoys), while TBP-TATA-box interaction controls sub-hour fluctuations (long permissive/non-permissive periods). A cellular promoter also produces polymerase convoys and displays multi-scale bursting. We propose that slow, TBP-dependent fluctuations are important for phenotypic variability of single cells.

## Abstract (web summary)

HIV-1 viral gene expression stochastically switches between active and inactive states. Here, using improved single molecule RNA microscopy, the authors show that HIV-1 RNA stochastic transcription is achieved by groups of closely spaced polymerases, and is regulated by Mediator and TBP at different time scales.

## Graphical abstract

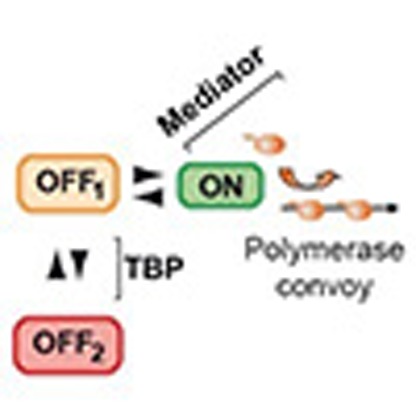

RNA synthesis is a fundamental step in gene expression. Transcription initiation is promoted by factors that recruit RNA polymerases at the promoter, melt DNA and load the template strand in the active site of the polymerase^1^,^2^,^3^. For RNA polymerase II (RNAPII), these steps require the combined action of the general transcription factors (GTF), which form the preinitiation complex (PIC). TFIID is the first GTF recruited to DNA and it recognizes the promoter sequences. It contains the TATA-binding protein (TBP^4^) and TAF1/2 that recognize the initiator element. Binding of TBP is a key step and an important target for gene regulation^5^,^6^. TFIIA stabilizes TBP binding at promoters, while BTAF1 dissociates it. TFIIB, TFIIE, TFIIF and TFIIH are recruited following TFIID binding^1^,^2^,^3^. They play direct roles in the initiation process by interacting with the polymerase and the promoter DNA. In particular, TFIIH triggers promoter melting. It also phosphorylates RNAPII on the serine 5 of its CTD repeats, and this facilitates promoter clearance^7^. RNAPII then often makes a promoter-proximal pause. This pause is released by the kinase CDK9 that phosphorylates negative elongation factors and RNAPII CTD on its serine 2 (ref. 7). In the case of HIV-1, CDK9 is recruited by the viral transactivator Tat bound to nascent viral RNAs^8^.

Another key player is the Mediator^9^,^10^. It was initially characterized as a factor required for the stimulation of transcription by transcriptional activators, but later shown to be also required for basal transcription. It binds to RNAPII–TFIIF complexes and to several GTFs such as TBP, TFIIE and TFIIH. These interactions and the analysis of yeast mutants suggest that Mediator recruits RNAPII and PIC components to the promoter. In addition, Mediator stimulates the kinase activity of TFIIH, and *in vitro* experiments have shown that Mediator stimulates reinitiation by maintaining TBP, TFIIE and TFIIH at the promoter following RNAPII escape^11^,^12^. A simple model of transcription activation thus involves binding of trans-activators to upstream enhancers, chromatin remodelling at promoters, and recruitment of GTFs and RNAPII through the Mediator.

Transcription initiation has been intensively studied *in vitro*, and more recently, directly in living cells. Within the cellular environment, promoter activity undergoes stochastic fluctuations, commonly referred to as transcriptional noise^13^,^14^,^15^,^16^. These fluctuations occur in various organisms and translate into cell-to-cell differences in gene expression. Consequently, they contribute to phenotypic variability^17^,^18^. In particular, experiments with green fluorescent protein (GFP)-tagged HIV-1 viruses showed that viral gene expression stochastically switches between active and inactive states, with the active state being maintained over days by a Tat-dependent positive-feedback loop^19^,^20^,^21^. Stochastic inactivation of the viral promoter is likely important for entry into latent states. Likewise, stochastic, inefficient activation of latent viruses is essential for maintaining the viral reservoir in patients^22^. Characterizing the factors controlling fluctuations of promoter activity is thus important for HIV-1 therapies.

Transcriptional noise has been observed using GFP-tagged proteins, single-molecule fluorescent *in situ* hybridization (FISH; smFISH) and direct tracking of promoter activity using MS2-tagged RNAs^14^,^16^,^23^. In eukaryotic cells, it is believed that a major source of noise lies in the dynamic binding of transcription factors and nucleosomes at the promoter, which may trigger random switching between active and inactive states. This model has, however, not been extensively tested, and given the complexity of the transcription initiation machinery, it is currently unclear how all the factors involved impact transcriptional noise. A persistent technical limitation is the difficulty to measure transcriptional activity in live cells with single polymerase sensitivity, high temporal resolution and long time scales. Here we overcame this limitation by improving RNA-tagging technologies. This allowed us to characterize HIV-1 transcription with high precision and to analyse the role of the basal transcription machinery in generating transcriptional noise. In particular, we show that the HIV-1 promoter generates polymerase convoys: groups of closely spaced polymerases that elongate together through the gene body. Polymerase convoys arise by a Mediator-dependent reinitiation mechanism and are also observed for a cellular promoter. We further show that the HIV-1 promoter fluctuates on two time scales, minute and sub-hour, which are independently regulated by distinct factors. TBP/TATA box interaction regulates the rate of slow fluctuations, while Mediator controls the rapid fluctuations. Different promoter elements thus appear to impact noise at different time scales.

## Results

### Improving MS2 labelling to track single RNAs in live cells

**Figure 1:**
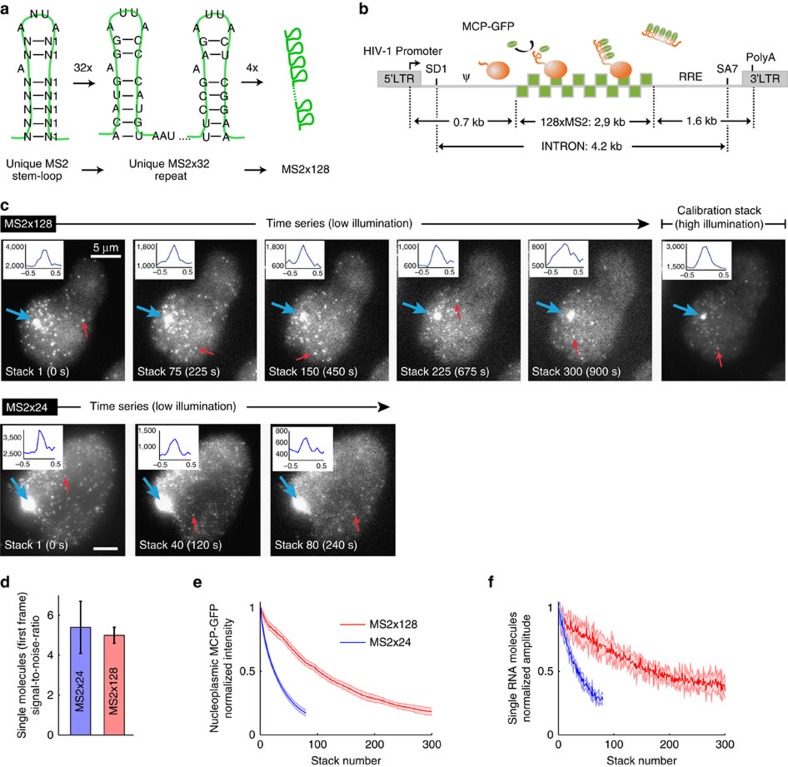
Characterization of the MS2x128 RNA tag. (**a**) Generation of the MS2x128 RNA tag. (**b**) Schematic of the HIV-1 reporter construct. The green squares represent the MS2 repeat; the green oval, MCP-GFP; the orange ball, RNAPII with the nascent RNAs. LTR: the HIV-1 long terminal repeat; SD1: the major HIV-1 splice donor; SA7: the last splice acceptor; Ψ: packaging sequence; RRE: Rev-responsive element. (**c**) Comparison of the MS2 × 24 and MS2 × 128 tags. Images are selected planes from an epifluorescence 4D stack and show cells expressing the MS2-tagged reporter genes. Excitation power was set to obtain comparable signal-to-noise ratios (SNR) for the two reporters in the first image stack. SNR is the mean signal of single molecules divided by the s.d. of the background. Top right panel: a calibration stack was recorded at the movie end with a higher excitation power (see Methods section). All images were rescaled to display similar intensities for single molecules. Red arrows: individual RNA molecules. Blue arrow: transcription site (TS). Signal saturation is due to rescaling. Inset: intensity line profile through single-molecule spots. Scale bar, 5 μm. (**d**) SNR of single RNA molecules. Plot shows mean values and standard deviations of SNR of single RNA molecules in the first frame of movies, for the MS2 × 24 and MS2 × 128 tags (6 cells; >500 single RNA molecules). (**e**) Bleaching of MCP-GFP. Graph shows normalized intensity of nucleoplasmic MCP-GFP as a function of the frame number (mean and s.d.). Intensities were averaged over the nucleoplasm and normalized to the first frame (five cells). (**f**) Intensities of single RNA molecules. Graph shows estimated amplitude of 3D Gaussians that fit isolated RNA molecules (mean and s.d.), after normalization to the first frame (five cells; >500 single molecules). Reliable fits could no longer be obtained after 80 stacks for MS2 × 24.

We previously developed a widely used system to visualize single messenger RNAs (mRNAs) in live cells^24^,^25^,^26^. It is based on the insertion of 24 MS2 binding sites in the gene of interest and on the expression of a fusion between GFP and the MS2 coat protein (MCP), which recognizes these repeats on the synthesized RNA. This approach detects single molecules, but requires a strong illumination power that leads to rapid photobleaching. Here we designed a novel MS2 tag with a higher number of binding sites. To improve folding and to prevent plasmid instability, we created 32 distinct MS2 stem loops, each predicted to bind the MCP protein with high affinity^27^ (Fig. 1a). This MS2x32 sequence was then duplicated to generate repeats of 64 and 128 stem loops. Due to unwanted mutations, only 120 stem loops were functional (see Methods section).

We constructed an HIV-1 reporter gene that contained the 5′ and 3′ LTRs harbouring the viral promoter and polyA sites, respectively^28^ (Fig. 1b). It also comprised the major HIV-1 splice donor SD1, the last splice acceptor SA7, and the packaging sequences and Rev-responsive element elements. The novel MS2x128 repeat was inserted in the resulting intron to avoid export of such a large artificial RNPs, and a single copy of the reporter was introduced in HeLa cells using the Flp-In recombination system. Expression was dependent on the viral transactivator Tat (Supplementary Fig. 1A). Northern blot analysis of RNAs extracted from an Actinomycin D time course indicated that the pre-mRNA spliced with a lifetime of 45 min (Supplementary Fig. 1B). In agreement with a slow splicing rate, reverse transcription PCR (RT–PCR) and smFISH showed that splicing was entirely post-transcriptional, also consistent with previous reports analysing HIV-1 splicing^28^,^29^ (Supplementary Fig. 1C). Live-cell observations showed a rapid appearance and disappearance of signals at the transcription site (Supplementary Movies 1 and 2). This indicated an absence of retention of unspliced RNA at the gene, which is likely due to a lack of co-transcriptional assembly of the splicesome. Thus, by measuring the intensity of the transcription sites (TS) over time, we could measure promoter activity without significant contribution of the splicing reaction.

Stable expression of MCP-GFP allowed excellent visualization of MS2x128 tagged pre-mRNA in fixed samples (>85% of single-molecule co-detected by smFISH; Supplementary Figure 1D–1E). We then compared MS2x128 and MS2x24 in time-lapse movies, adjusting illumination conditions for each tag such that single pre-mRNA molecules had similar intensities and signal-to-noise ratios in the first image stack (Fig. 1c,d). Because of the lower illumination power required for the MS2x128 reporter cells, the resulting photobleaching was approximately four times slower with this tag than with MS2x24. This allowed for reliable single-molecule detection over 3,000 two-dimensional images for MS2x128, instead of 600 for MS2x24 (Fig. 1e,f). We concluded that the MS2x128 tag provides reliable RNA detection and allows single-molecule visualization through hundreds of time points in three-dimensions (3D).

### Tat-activated HIV-1 promoters yields polymerase convoys

**Figure 2:**
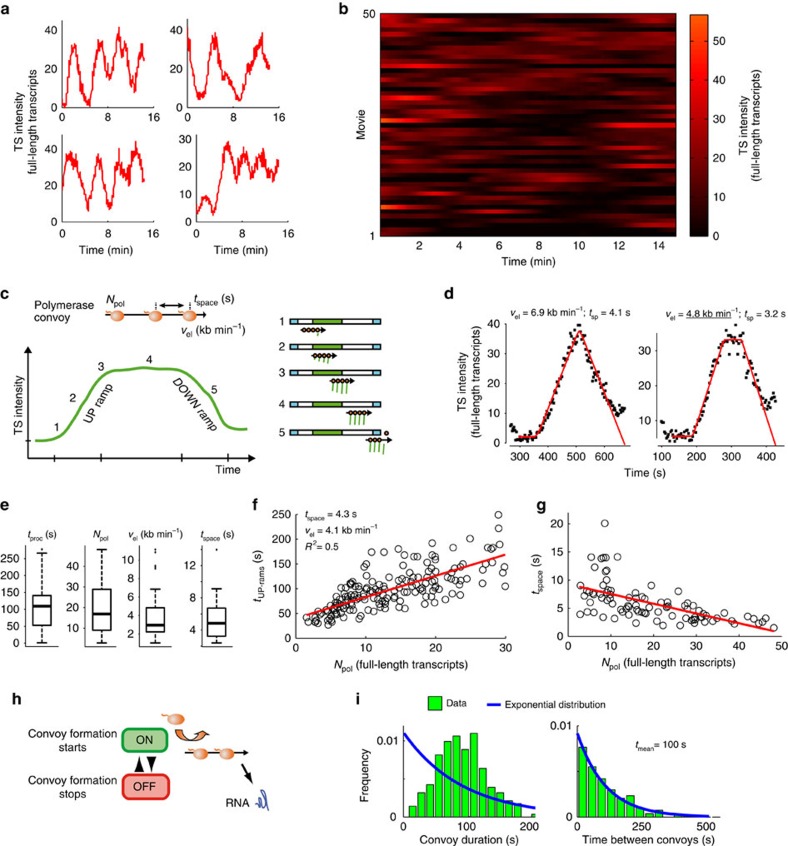
Kinetic parameters of HIV-1 polymerase convoys in *High Tat* cells. (**a**,**b**) TS intensities over time. In **a**, *x* axis is time in s and *y* axis is the intensity of TS, expressed in equivalent number of full-length RNA molecules. In **b**, each lane is a cell and the TS intensity is colour coded (scale on the right). (**c**) Transcription by polymerase convoys. Top schematic of a polymerase convoy. *N*_pol_: number of polymerase; *t*_space_: RNAPII spacing (in s); *v*_el_: elongation rate. Bottom and right: schematics describing the different phases of a transcription cycle. (**d**) Fits of isolated transcription cycles with the polymerase convoy model. Black dots display experimental values of TS intensities (in number of full-length pre-mRNA molecules) as a function of time (in s). Red curves show best fit to the model. *v*_el_: estimated elongation rate; *t*_sp_: spacing. Underlined number: only a minimum value was estimated for *v*_el_. (**e**) Box-plots representing the parameter values of the best-fit models, measured for a set of 90 isolated transcription cycles. Bottom dotted line displays the first quartile, the box corresponds to the second and third quartile, the top dotted line to the last quartile, and the horizontal line to the median. Small circles are outliers (1.5 times the inter-quartile range above or below the upper and lower quartile, respectively). (**f**) Estimation of mean values of *t*_space_ and *v*_el_ from a regression analysis of pooled UP ramps. Graph displays duration of UP ramps as a function of *N*_pol_. Each circle is an UP ramp. Red line is the linear trend. (**g**) Correlation between *t*_space_ and *N*_pol_. Each isolated transcription cycle is represented by a black circle. Red curve shows a linear fit of the data. (**h**) Schematic indicating how stochastic ON/OFF switching of a promoter creates polymerase convoys. (**i**) Histograms of the duration of individual convoys and time intervals between convoys. Data are from isolated transcription cycles. Convoy duration is estimated by multiplying N_pol_ with *t*_space_, and time between convoys is the time between the end of the UP ramp of an isolated transcription cycle and the beginning of the next UP ramp (t_mean_ is the average).

We first analysed HIV-1 transcription when the promoter was fully activated, by expressing the viral trans-activator Tat constitutively and at saturating levels (*High Tat* cells). Saturation was demonstrated by the fact that further increasing of Tat levels by >20-folds only marginally increased pre-mRNA levels (Supplementary Fig. 2). Since Tat releases promoter-proximal pausing^6^, this step is thus not rate limiting in our reporter system. We imaged cells for 15 min at a rate of one 3D stack every 3 s. We refer to this acquisition condition as short movies. Intensities of TS varied greatly during these movies, indicating both active transcription initiation and transcript release (Fig. 2a,b; Supplementary Movies 1 and 2). In about 25% of the movies, TS transiently turned off. These short OFF periods were often followed by an isolated intensity peak, formed by a rapid signal increase and decrease. These intensity peaks were often substantially brighter than single molecules in the nucleoplasm. Therefore, they likely corresponded to initiation, elongation of polymerases and release of transcripts. In the following, we refer to these peaks as isolated transcription cycles.

One limitation of this analysis is that the number of engaged polymerases at the TS is not known. We thus developed an image analysis pipeline that allowed absolute quantification of the number of pre-mRNA at the TS (Fig. 2b; for details see Supplementary Notes and Supplementary Fig. 2). This approach is based on the acquisition of an additional image stack—termed calibration stack—immediately at the end of the movie with higher illumination power. The increased quality of this stack allowed quantifying single-RNA-molecule intensities in the nucleoplasm. The averaged RNA intensity was then used to infer the number of full-length transcripts at the TS. The quantification pipeline was robust since in validation movies, the intensities measured for single nucleoplasmic RNA molecules was 1.1±0.3 across the movie (Supplementary Fig. 2). Quantification of TS intensities with this approach indicated that isolated transcription cycles involved a large number of RNA polymerases (Fig. 2a). These polymerases thus initiated in a relatively short time, typically a minute, and elongated together through the gene. This behaviour implies a tri-phasic curve for TS intensities (Fig. 2c). First, an increasing phase when new polymerases enter the MS2 repeat (UP ramp); second, a plateau when all the polymerases have passed the MS2 tag; and finally a decreasing phase when pre-mRNAs are 3′-end processed and released from the transcription site (DOWN ramp). Note that polymerases located before the MS2 repeat are not visible. A noticeable feature of the experimental curves was that the UP and DOWN ramps were linear (Fig. 2a,d; Supplementary Fig. 3A,F). This indicates important properties for the polymerases, which do not depend on mathematical modelling and can be directly inferred from the experimental data (Supplementary Note 1). A linear DOWN ramp indicated a regular release of pre-mRNAs, which could be explained by polymerases reaching the polyA site at regular time intervals. A linear UP ramp implied a constant synthesis rate of MS2 stem loops, indicating a stable and uniform speed for the polymerases. Thus, isolated transcription cycles are produced by a set of closely spaced polymerases that move at a constant speed, which we refer to as polymerase convoy.

### Quantitative parameters of HIV-1 polymerase convoys

We developed a mathematical model describing TS intensity as a function of the progression of the individual polymerases forming a polymerase convoy, assuming regular polymerase spacing for simplicity. This model contains four key parameters (Fig. 2c): the number of polymerases in a convoy (*Npol*), their spacing (*t*_space_, in s), their elongation rate (*v*_el_, in kb min^−1^), and the time for 3′-end processing and release from the TS (*t*_proc_, in s). We implemented analysis tools to estimate these parameters from the isolated transcription cycles (Supplementary Notes 1–3). Simulations accounting for realistic measurement errors indicated that correct parameter values could be recovered for convoys having not only the assumed regular spacing, but also a stochastic spacing (Supplementary Note 3, Supplementary Table I). Our simplified model of convoys thus accommodates different situations for polymerase spacing. Fitting the intensity curves for a total of 90 isolated transcription cycles yielded well-constrained parameters except for the elongation rate, for which only a minimal value could be determined in 60% of the cases (Fig. 2d and Supplementary Fig. 3I). We estimated mean values of 19 polymerases per convoy, with a spacing of 4.1 s, an elongation rate of 4.1 kb min^−1^, and a 3′-end processing/release time of 103 s (Fig. 2e). These values were confirmed by a linear regression analysis of the pooled UP ramps, which yielded similar mean elongation rate and spacing time (Fig. 2f; Supplementary Fig. 3J and Supplementary Note 1). Overall, our data indicate a residency time of 169 s for the nascent RNAs, consistent with previous MCP-GFP FRAP data with a similar HIV-1 reporter^28^. An elongation rate of 4 kb min^−1^ is also in range with previous measurements by microscopy and corresponds to the fastest genes in genome-wide GRO-Seq measurements^28^,^30^,^31^,^32^,^33^. Our measurements also predict that polymerases transcribe up to 6 kb down the polyA site, and this agrees with RNAPII ChIP-Seq measurements on mammalian genes^34^,^35^. Interestingly, analysis of the fitted parameters indicated a negative correlation for *Npol* and *t*_space_ (Fig. 2g). Convoys with more polymerases are thus more compact. This indicated that initiation rates could vary between convoys, perhaps due to specific microenvironments of the promoter.

The succession of polymerase convoys indicates that the promoter has a discontinuous activity and rapidly switches between ON and OFF states (Fig. 2h). The duration of ON periods corresponds to the time required to initiate all the polymerases of a convoy (the number of polymerases multiplied by their spacing), and OFF periods to the time separating two convoys (Supplementary Fig. 3K). We found that the time separating two convoys followed a single exponential distribution with a time constant of 100 s, indicating that convoy initiation may be driven by a single rate-limiting step (Fig. 2i). In contrast, the duration of individual convoys were not fit by an exponential distribution, suggesting more complex, multi-step kinetics to turn the promoter off.

### Mediator controls the formation of polymerase convoys

**Figure 3:**
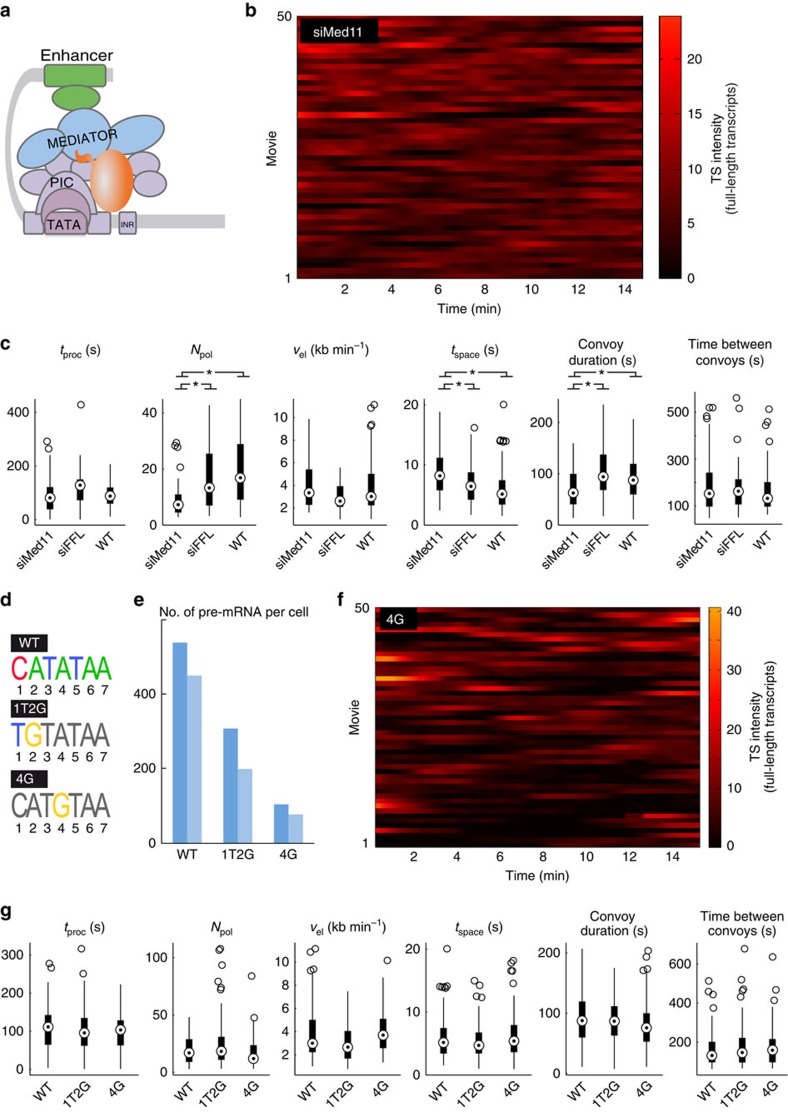
Effect of Mediator and TATA box on HIV-1 transcription and on short time scales. (**a**) Schematic of an initiating polymerase. Mediator is in blue; GTFs in violet; upstream enhancers and bound factors in green; RNAPII in orange. (**b**) Intensities of TS over time. Each lane is a cell and TS intensity is colour coded, and expressed in equivalent number of full-length RNA molecules. Note that the scale is different than in **f** and Fig. 2b. (**c**) Analysis of convoy parameters in control cells or in cells lacking a functional Mediator complex. Box-plots display the convoy parameter distributions obtained by fitting individual transcription cycles as in Fig. 2d, using cells treated with siRNAs targeting Med11 (siMed11) or firefly luciferase as control (siFFL). Untreated wild-type reporter cells: WT. Asterisks: *P* value <0.05 (KS test, *n*>36). Data are from short movies. (**d**) Sequences of the HIV-1 TATA box and its mutants. (**e**) Expression of HIV-1 pre-mRNAs in the WT and mutant reporters. Values are numbers of nucleoplasmic pre-RNAs per cell, as detected by smFISH, and are shown for two independent clones. Dark blue: the clones used for live-cell analysis. (**f**) Intensities of TS over time. Each lane is a cell and TS intensity is colour coded, and expressed in equivalent number of full-length RNA molecules. (**g**) Convoy parameters in HIV-1 WT and mutant promoters. Box-plots display the distribution of the parameters obtained by fitting isolated transcription cycles with the polymerase convoy model (*N*>100 for 1T2G and 4G; *N*>90 for WT). Data are from short movies.

We then sought to identify factors controlling convoy formation and focused on Mediator. Mediator contacts many PIC components in addition to RNAPII (refs 9, 10; Fig. 3a), and it is therefore ideally placed to promote the rapid succession of initiation events required to produce convoys. Knockdowns of a number of Mediator subunits have been shown to affect basal and Tat-activated HIV-1 transcription without affecting cell viability^36^,^37^,^38^. In agreement, MED11 knockdown reduced nascent HIV-1 RNA levels by more than twofold (Supplementary Fig. 4A–C). Analysis of short movies showed that convoy formation was significantly affected in these knockdowns. The mean number of polymerases per convoy was reduced from 19 to 8 (Fig. 3b; note that the colour scale of Fig. 3b is narrower than Fig. 2b and Fig. 3f), and we observed an increased spacing between polymerases (from 4 to 8 s, *P*<2 × 10^−6^ with a KS test; Fig. 3c). The promoter ON time was also reduced from 90 to 68 s (*P*<2 × 10^−4^ with a KS test). Thus, Mediator was important for the rapid succession of initiation events occuring during convoy formation.

### The TBP/TATA box interaction controls long ON/OFF periods

**Figure 4:**
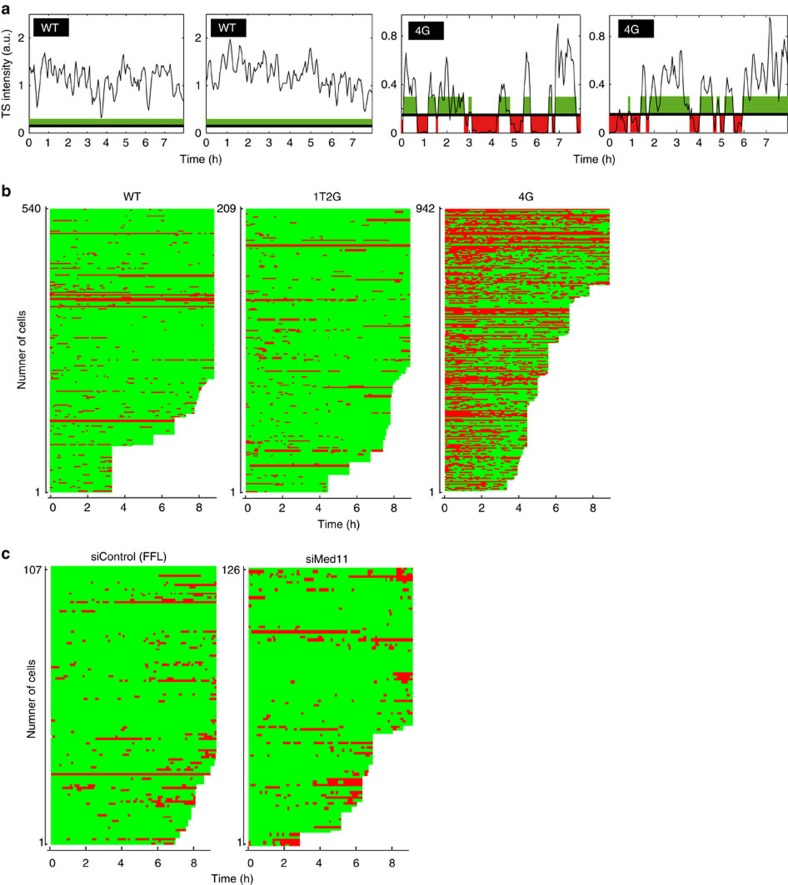
Effect of Mediator and TATA box on HIV-1 transcription and on long time scales. (**a**) Fluctuation of TS insensities over long time scales, for WT and 4G TATA mutant. Graphs display the integrated fluorescence intensity of individual TS recorded over 8 h (in a.u.), with one image stack recorded every 3 min (long movies). Time scale is in hours (*x* axis). Green bars: permissive periods; red bars: non-permissive periods. One panel is one cell, with the reporter name indicated in a black box. (**b**) Distribution of permissive and non-permissive periods of many TS, for HIV-1 WT and TATA box mutants. Graphs show the permissive and non-permissive periods in green and red, respectively, with each line being an individual cell. The *x* axis is time, in h. The name of the reporter is indicated on the top. (**c**) Distribution of permissive and non-permissive periods for control and MED11 knocked-down cells. Legend as in **b**.

TFIID binding is a fundamental event in PIC formation^5^,^6^. We thus next focused on TBP/TATA box interaction. We generated two reporters with altered TATA sequences, 1T2G and 4G (Fig. 3d), which were previously shown to decrease HIV-1 expression by three- and fivefold, respectively^39^. Furthermore, it has been shown that *in vitro*, these mutations decrease the affinity for TBP by two- and ninefold, respectively^40^, indicating that the activity of the HIV-1 promoter correlates the affinity of TBP for the TATA box. We integrated these constructs at the same genomic location as the wild-type (WT) reporter, using the Flp-In site-specific recombination system. SmFISH experiments showed that the WT reporter expressed on average 492 molecules of pre-mRNA per cell, while 1T2G expressed 252, and 4G only 90, with little variability between independent Flp-In clones (Fig. 3e). We then recorded short movies and measured the parameters of polymerase convoys. We found no significant differences between reporters, with a similar number of polymerases per convoy, elongation rates, polymerase spacing, convoy duration and time between convoys (Fig. 3f,g). This was unexpected for 4G as it expresses nearly fivefold less pre-mRNAs than WT. We reasoned that this lower level might result from a different behaviour on longer time scales. We thus recorded movies for up to 8 h, using a lower temporal resolution of one 3D stack every 3 min (acquisition conditions referred to as long movies). While TS of WT cells remained active nearly all the times, we found that the TS of 4G cells showed frequent periods of inactivity lasting minutes to hours, which alternated with period of activity lasting tens of minutes (Fig. 4a,b, Supplementary Movies 3 and 4). Since many convoys were fired during the active periods, we refer to them as permissive, while the long inactive periods are referred to as non-permissive. Non-permissive periods were fit with a bi-exponential function with lifetimes of 9 min (77% of the events) and 34 min (23% of the events; Supplementary Fig. 5A,B). Most non-permissive periods were thus too long to be observed in the short movies that focused on actively transcribing cells. Interestingly, non-permissive periods were also observed for the WT and 1T2G reporters, albeit infrequently (Fig. 4b). In these cases, the duration of non-permissive periods showed a bi-exponential distribution very similar to that observed for the 4G reporter, but they were five times less frequent (Supplementary Fig. 5B). These experiments thus revealed that the HIV-1 promoter displays infrequent periods of prolonged inactivity, and that the 4G mutation specifically increases their probability. Since the 4G mutation weakens the interaction with TBP by about ninefold^40^, these non-permissive periods are likely due to the dissociation of TBP from the promoter.

### Quantitative modelling of rapid and slow fluctuations

**Figure 5:**
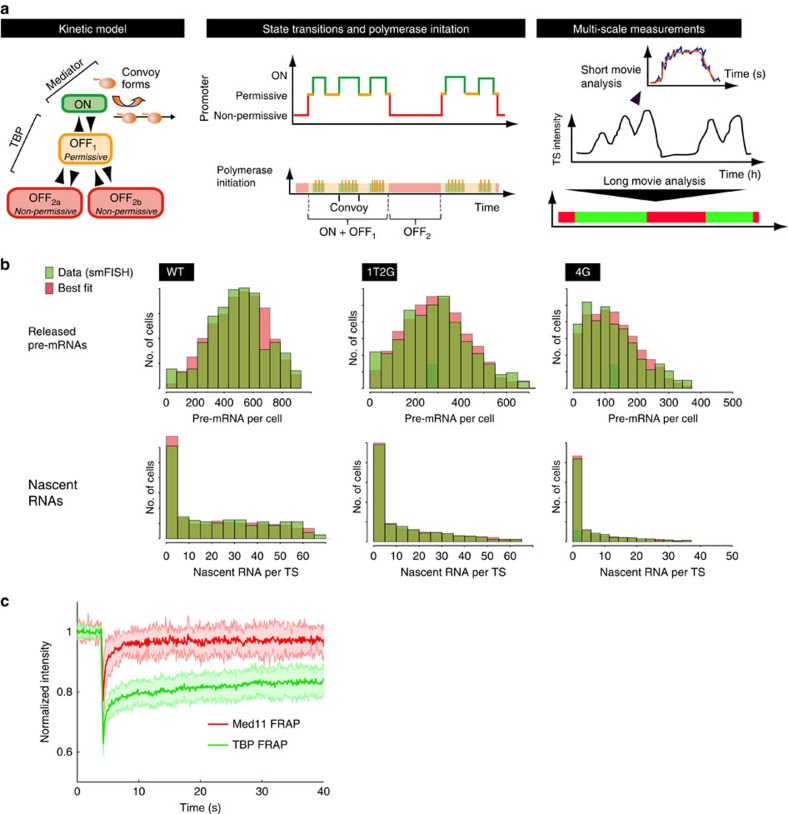
Modelling of the distribution of mature and nascent HIV-1 RNA in *High Tat* cells. (**a**) Kinetic scheme of the promoter states identified in live-cell experiments. Left: schematic of a model with three promoter states accounting for permissive and non-permissive periods. Middle: schematic describing promoter state over time (top), and corresponding polymerase initiation events (bottom). Right: TS intensity measurements (middle) and the corresponding data extracted from short movies (top), or long movies (bottom). (**b**) Best-fit simulated distribution of the number of released and nascent pre-mRNA per cell, for the different reporters. Experimental RNA distribution are from smFISH data with the indicated reporter (green) and best-fit distributions are from the model of **a** (red). See Supplementary Fig. 6 for parameter values. (**c**) FRAP analysis of the dynamic of GFP-TBP (green) and GFP-MED11 (red) in the nucleoplasm of HeLa cells. Numbers represent the mean fluorescent intensities of the bleached spot (1.3-μm diameter) over time, after photobleaching correction and normalization to pre-bleach values (*n*>12;±s.d.).

A typical permissive period contained several polymerase convoys, and these convoys were separated by short OFF periods (Fig. 2a,b). Several lines of evidence indicate that the short OFF periods that separate two convoys have a different nature than that the non-permissive periods. First, non-permissive periods were significantly longer than the time separating two convoys (9 or 34 min versus 1.5 min; compare Supplementary Fig. 5B and Fig. 2i). Second, the TATA box mutation selectively increased the frequency of non-permissive periods and did not affect the time separating convoys (Supplementary Fig. 5A, insets, and Fig. 3g). Third, knocking down Mediator selectively impacted convoys, and had no effect on the frequency or duration of non-permissive periods (Fig. 4c). The different nature of these two inactive periods indicates that the HIV-1 promoter has two distinct OFF states (see model in Fig. 5a): one that generates interruptions between convoys (OFF_1_, dependent on Mediator), and one that creates non-permissive periods (OFF_2_, dependent on the strength of the TATA-box).

Within a clone, cell-to-cell variations in gene expression are believed to be due to stochastic fluctuations of promoter activity^23^. Indeed, when we simulated the model of Fig. 5a with parameter values in range with those estimated from our live-cell data, it could reproduce the cell-to-cell variability in RNA levels, as measured by smFISH (Fig. 5b; Supplementary Fig. 6). This indicated that the major sources of fluctuations are captured by the live-cell data and are correctly described by the model. Taken together, these data indicate that the HIV-1 promoter fluctuates on two distinct time scales, minutes to produce polymerase convoys, and sub-hour to generate permissive/non-permissive periods. We refer to this phenomenon as multi-scale bursting.

A common hypothesis is that transcriptional noise originates from the stochastic binding of transcription factors at promoters^23^. According to this model, the duration of a binding event should determine the duration of an active period. To test this idea, we performed FRAP experiments in the nucleoplasm of cells expressing fluorescently-tagged TBP and MED11 (Fig. 5c). MED11 recovered rapidly and TBP recovered slowly. Given that Mediator affects rapid fluctuations and TBP slow ones, these FRAP data suggest that the time scale of the fluctuations are related to the exchange rates of a factor with DNA.

### A cellular promoter also produces polymerase convoys

**Figure 6:**
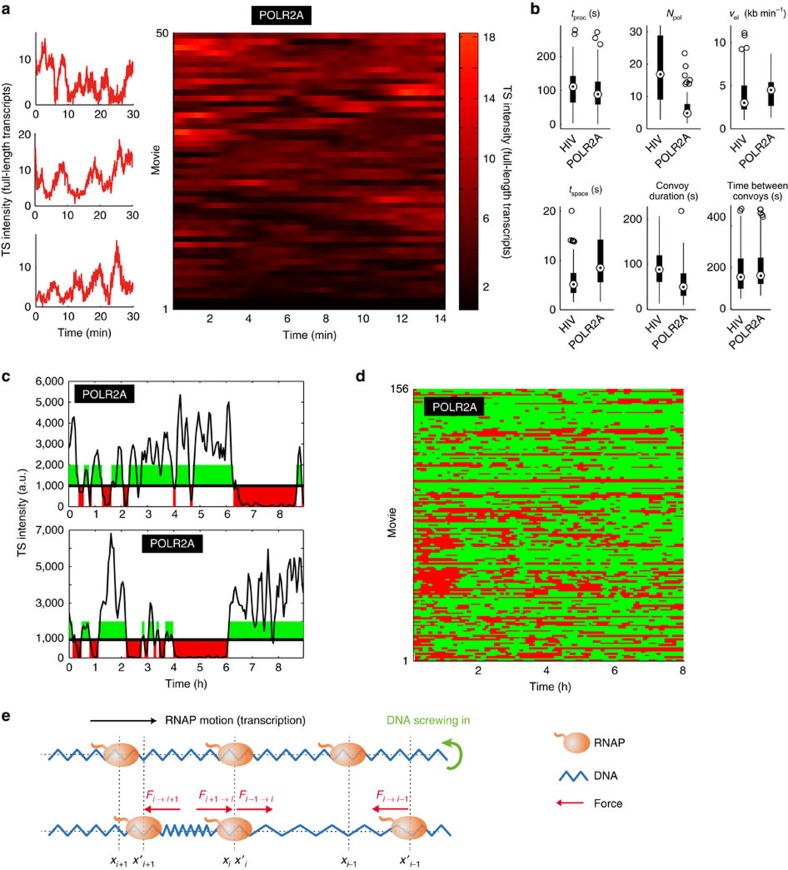
Kinetic analysis of the *POLR2A* promoter activity and model of polymerase convoy. (**a**,**b**) Activity of the *POLR2A* promoter analysed at high temporal resolution and short time scales. In **a,** left panels, *x* axis is time and *y* axis is the TS intensity, expressed in equivalent number of full-length RNA molecules. In **a,** right panel, each lane is a cell and the TS intensity is colour coded. In **b**, box-plots display the convoy parameter distributions obtained by fitting individual transcription cycles (legend as in Fig. 2e). Data are from short movies; time scale is in min. (**c**,**d**) Activity of the *POLR2A* promoter over long time scales. Graphs show the permissive and non-permissive periods displayed in green and red, respectively. (**c**) Graphs display the integrated fluorescence intensity of individual TS recorded over 8 h (in a.u.). (**d**) Each line is an individual cell. Data are from long movies; time scale is in h. (**e**) Physical modelling of an elongating polymerase convoy. Top: DNA screws into an elongating polymerase convoys. Bottom: a polymerase stops and generates supercoiling constraints with the preceding and succeeding polymerases. *F*_*i*_ indicates forces encountered by the *i*th polymerase.

Next, we determined whether polymerase convoys are specific for HIV-1 or can also be observed for cellular genes. To this end, we analysed the promoter of the *POLR2A* gene, a housekeeping gene coding for the large sub-unit of RNAPII. We cloned the corresponding promoter in the MS2x128 reporter construct in place of the HIV-1 promoter and inserted it in HeLa cells in a single copy using the Flp-in system. SmFISH showed that this is a moderate promoter, with 5.5 nascent pre-mRNAs per active TS, as compared with 32 for HIV-1 (Supplementary Fig. 7A,B). Analysis of splicing pattern showed that the reporter spliced post transcriptionally, similar to the HIV-1 construct (Supplementary Fig. 7C). Remarkably, short movies showed that the *POLR2A* promoter produced polymerase convoys (Fig. 6a). Fits of individual transcription cycles further showed that the *POLR2A* convoys contain fewer polymerases as compared with the HIV-1 promoter (6.5 versus 19), and that they have a larger spacing (8.5 versus 4 s; Fig. 6b). The specific characteristics of polymerase convoys thus appear to be promoter dependent. Analysis of long movies indicated that the *POLR2A* promoter displayed prolonged periods of inactivity, as seen with HIV-1 (Fig. 6c,d). On average, the promoter remained active for 30 min, during which time it generated a number of convoys, and was then inactive for 20 min (Supplementary Fig. 7D). Altogether, these data indicate that the *POLR2A* promoter produces polymerase convoys and fluctuates on two time scales, minutes and sub-hour.

## Discussion

By imaging transcription sites in live cells at high temporal resolution, we observed that the *POLR2A* and Tat-activated HIV-1 promoters generate polymerase convoys: group of closely spaced polymerases that elongate together through the gene body. Remarkably, the existence of polymerase convoys is supported by previously published electron microscopy images of chromatin spreads, also called Miller spreads^41^,^42^,^43^. Such electron microscopy images revealed ‘polymerase clusters', which correspond to sets of closely spaced RNAPII enzymes transcribing a DNA segment^41^,^42^,^43^. In both *Drosophila* and rat liver cells, a gene can contain several successive polymerase clusters, and in a remarkable study made more than 30 years ago, the gaps separating these clusters were predicted to result from brief interruptions in transcription initiation^43^. The number of polymerases in a cluster, the spacing between polymerases and the size of the gaps between clusters, as observed in electron microscopy images, are in good agreement with our live-cell measurements (Supplementary Table 2). Polymerase clusters and convoys thus likely correspond to the static and dynamic views of the same phenomenon: polymerases rapidly initiating and escaping the promoter one after the other, until the promoter stochastically switches into an inactive OFF state. Single, isolated polymerases are also observed on Miller spreads^41^. In the future, it will be interesting to determine which genes are transcribed by convoys or single polymerases. Current smFISH data often report bright transcription sites, which contain many pre-mRNA molecules^44^. This suggests that a sizeable percentage of mammalian genes might be transcribed by convoys.

The occurrence of polymerase convoys provides a basis to refine our views on the general phenomenon of gene bursting. In general, bursts are defined as inhomogeneous temporal distributions of initiation events, and are often modelled by stochastic ON/OFF switching of promoters. However, this notion does not specify a time scale and has been used to describe fluctuations occurring in minutes or hours (for instance, see refs 15, 45). We propose to define convoys as a burst having three distinctive properties: (i) a short time scale, with polymerases initiating one after the other every few seconds for about a minute; (ii) a reinitiation mechanism, with some GTFs remaining bound to the promoter during convoy formation (see below); (iii) shared properties between polymerases belonging to the same convoy, such as common initiation and elongation rates. These rates can, however, vary between convoys.

Monitoring transcription at time scales ranging from seconds to hours showed that the *POLR2A* and the Tat-activated HIV-1 promoters fluctuate on two distinct time scales: at the minute scale to produce convoys, and at the sub-hour scale to generate permissive/non-permissive transcriptional periods. The notion of burst is thus not precise enough to describe this situation, and instead we propose the term of multi-scale bursting. Importantly, different promoter elements selectively impact each time scale. Weakening the TATA-box only increases the frequency of long non-permissive periods, while decreasing Mediator levels only impacts convoys. FRAP experiments indicate that MED11 interacts much more transiently with DNA than TBP. This agrees with the idea that promoter fluctuations originate in the stochastic binding of transcription factors^23^, where proteins residing a long time generate slow fluctuations, and proteins residing a short time create rapid fluctuations.

Our FRAP data, as well as previously published ones^46^, indicate that TBP residency time on DNA is in the range of minutes, and thus significantly longer than the few seconds separating two initiation events during convoy formation. The HIV-1 promoter thus likely uses a reinitation mechanism, where TBP remains bound to the TATA box during the succession of initiation events that generates polymerase convoys. Reinitiation is a mechanism that was proposed early on^47^,^48^, and previous *in vitro* observations indicated that this process is facilitated by a TATA box and by Mediator^11^,^12^,^49^. Our *in vivo* data are consistent with these results since the HIV-1 promoter possess a strong TATA box and we found that Mediator promotes the rapid succession of initiation events occurring during convoy formation.

Recent measurements by GRO-Seq indicate that elongation rates vary between genes^30^, and also along the length of the same gene^32^. Our data further indicate that different convoys can have different velocities while transcribing the exact same sequence. Interestingly, single-molecule *in vitro* experiments have shown that even for T7 RNA polymerase, different molecules can adopt different velocities^50^. The reasons for these variations are currently poorly understood. However, given the known role of the elongation rate in regulation splicing and other RNA-processing steps, these variations could have profound functional consequences.

While our data suggest that different convoys have different velocities, it appears that polymerases within a convoy move at the same speed (Fig. 2e). On average, polymerases are separated by 280 nucleotides, and several mechanisms could explain a common speed in absence of physical contacts between polymerases. One that is supported by physical considerations involves DNA torsional forces. Transcription generates positive supercoiling ahead of the polymerase and negative one behind^51^,^52^. In the case of a convoy, no torsional stress is present within a convoy as long as polymerases elongate at the same speed (Fig. 6e). However, if a polymerase moves faster or slower than its neighbours, torsional stress will rapidly build up between them and will exert a counteracting force until all polymerases return to an identical elongation rate. We created a physical model of convoys and calculated that the torsional forces are strong enough to couple polymerases (Supplementary Note 4). While our data do not exclude other mechanisms, this model is supported by the recent data showing that DNA torsional forces regulate elongation rates in bacteria^53^. Calculations further indicated that the polymerase stall force scales with the number of polymerases. Convoys may thus elongate more efficiently than isolated polymerases. This agrees with the GRO-Seq data indicating that polymerases move faster on genes transcribed more intensely^30^,^32^.

Gene expression noise can have important phenotypic consequences^17^,^18^. In case of HIV-1, it is believed that transcriptional noise can act as a stochastic switch to direct infected cells into acute or latent states. Indeed, experiments with GFP-tagged viruses showed that infected cells can switch between ON and OFF states, with the ON state being maintained over days by the positive Tat feedback loop^21^. The stochastic switching between ON and OFF states is believed to arise from fluctuations in the levels of Tat, themselves due to transcriptional noise at the level of the HIV-1 promoter. A recent study has further shown that weakening the HIV-1 TATA box promotes a faster rate of switching between GFP expressing and non-expressing states^19^. This is consistent with our results that a TATA-box mutation increases the probability of long inactive periods, even in the presence of Tat. These long periods of inactivity may deplete Tat from cells, allowing inactivation of the positive-feedback loop.

Studies in yeast using GFP-tagged proteins have also revealed that TATA-containing, inducible genes are noisier^54^, and that the TATA box contributes to high noise levels^17^. The effect of the TATA box on slow fluctuations might thus extend beyond HIV-1, and the stochasticity of TBP binding could be a major determinant of phenotypic variability, as opposed to other PIC components producing short-term fluctuations that may be buffered by the RNA and protein half-lives.

## Methods

### Cells

HeLa Flp-in H9 cells (a kind gift of S. Emiliani) were maintained in DMEM supplemented with 10% fetal bovine serum, penicillin/streptomycin (10 U ml^−1^) and glutamin (2.9 mg ml^−1^), in a humidified CO_2_ incubator at 37 °C. Cells were transfected with the indicated plasmids or siRNAs for 48 h with JetPrime (Polyplus), following manufacturer recommendations. SiRNAs against Med11 had the following sequences (only one strand is shown): 5′-GAGAAUUCCCAGAGUGAUAdTdT-3′; Control siRNAs were 5′-CAACAGAAGGAGAGCGAAAdTdT-3′ and siRNA targeting Firefly Luciferase.

Stable expression of MCP-GFP was achieved by retroviral-mediated integration of a self-inactivating vector containing an internal ubiquitin promoter^55^. The MCP used dimerizes in solution and contained the deltaFG deletion, the V29I mutation, and an SV40 NLS^24^,^56^. MCP-GFP expressing cells were grown as pool of clones and FACS-sorted to select cells expressing low levels of fluorescence. Isogenic stable cell lines expressing the HIV-1 reporter gene were created using the Flp-In system and a HeLa H9 strain expressing both Tat (see below) and MCP-GFP. Flp-In integrants were selected on hygromycin (150 μg ml^−1^). For each construct, several individual clones were picked and analysed by *in situ* hybridization. Clones usually looked similar, and two of them were further selected for single-molecule RNA counting to measure clonal variability (20% for the TATA mutants, Fig. 3e). One clone was then further characterized by western and northern blotting, and used for the live-cell experiments.

High Tat cells were created using the plasmid pSpoII-Tat. In this plasmid, the CMV promoter transcribes a Tat-Flag cDNA followed by an IRES-Neo selectable marker. Following Neomycin selection (400 μg ml^−1^), expression levels of individual clones were verified by western blotting and by immunofluorescence to ensure homogeneity both between clones and between cells of a clone. Clone verification was additionally performed at every subsequent subcloning of the cell lines.

Saturating Tat cells were created by CRISPR genome-editing using a AAVS1 repair vector expressing Tat-Flag directly under the control of the chicken beta-actin promoter. Western blotting and smFISH against the MS2 repeat indicated that while the levels of Tat-Flag were 20 times higher in Saturating Tat cells than in High Tat ones, expression levels of the HIV-1 reporter were similar (Supplementary Fig. 2). This indicates that Tat levels are already saturating in High Tat cells.

### Plasmids

Sequences of the plasmids are available upon request. The novel repeats of the MS2 binding sites were cloned from chemically-synthesized oligonucleotides into pMK123 (ref. 57). The MS2 stem loops were separated by a linker of only three nucleotides, making the new repeat about twice as compact as the original one. The sequences of the 32 stem loops were designed to minimize their similarities, and to keep an optimal folding for each stem loop within the context of the repeat, using Mfold as a tool to assess the resulting RNA secondary structure^58^. The fact that the 32 stem loops have different sequences is expected to improve RNA folding, since when the stem loops have the same sequences, they can cross-hybridize between themselves and thus not fold as stem loops anymore. Also note that the stem loops do not have the C–>U mutation at position 3 of the loop, which is known to stabilize MCP binding by nearly 100-fold^59^. These may prevent stabilization of RNA degradation products in presence MCP as recently shown in yeast^60^. The 32 repeat was then multimerized to generate repeats of 64 and 128 stem loops, and cloned into the intron of an HIV-1 vector, which also contained an FRT-Hygro cassette for Flp-in recombination^28^ (see Supplementary Note 5 for cloning details). This generated the plasmid pIntro-MS2x128.

The HIV-1 TATA mutants were generated by first subcloning an ApaI–BamHI fragment containing the HIV-1 promoter into pBluescript-SK, then mutating the TATA element using the QuickChange random-mutagenesis kit (Stratagene), and cloning back the mutant promoters into pIntro-MS2x128.

The *POLR2A* reporter was constructed by exchanging the HIV-1 promoter (−453 to +55) for that of the mouse *POLR2A* gene (−624 to +91). This removes TAR and all HIV-1 upstream activating sequences but leaves most of the first HIV-1 exon.

### FRAP experiments and analysis

FRAP was performed on a confocal microscope (Meta LSM510; Carl Zeiss MicroImaging, Inc.) with a × 100∼ numerical aperture (NA) 1.4 objective. Nucleoplasmic GFP signal was bleached at 488 nm in a circle of 1.3-μm diameter at full laser power and for 300 ms. Recoveries were measured at a high frame rate (one image every 15 ms) and for 40 s using low power with the 488-nm laser line. Images were analysed as previously described^28^. Background was removed, intensities at each time point were corrected for bleaching by dividing them by the total cell fluorescence, and these values were finally normalized by dividing them with the fluorescent intensity before the bleach.

### RNA analyses and smFISH

Northern blots and competitive RT–PCR were performed according to standard procedure^61^. To assess co-transcriptional splicing, RT was primed with either oligo-dT or with an oligonucleotide-binding downstream the polyA cleavage site (sequence 5′-GCTGCTAGAGATTTTCCA-3′). Competitive PCR was performed with three oligos that amplified two short PCR products sharing one oligo, and which were specific for either the spliced or unspliced RNA (Supplementary Fig. 1B). Sequences were: 5′-AATGGGCAAGTTTGTGGAATTGGTT-3′; 5′-GATACCGTCGAGATCCGTTCA; 5′-CGAACAGGGACTTGAAAGCGA-3′.

For smFISH cells were fixed in 4% paraformaldehyde, permeabilized in 70% ethanol and hybridized with a mix of 10 fluorescent oligos^28^,^57^. Fluorescent oligos were directed against 32xMS2 repeats and each oligo contained four molecules of Cy3. Each oligo hybridized 4 times across 128xMS2 repeats, allowing for binding of 40 probes to single pre-mRNA molecule, thereby providing excellent single-molecule detection and signal-to-noise ratios (Supplementary Fig. 1D,E).

### Acquisition and analysis of smFISH images

To obtain the number of released, nucleoplasmic pre-mRNA per cell and their distribution in the cell population, smFISH images were recorded with an OMX Deltavision microscope in SIM mode. Acquisition was performed in 3D with a *z*-spacing of 0.125 μm, with a × 100 objective, and × 2 intermediate lens and an Evolve 512 × 512 EMCCD camera (Photometrics). Following reconstruction, images were analysed with FISH-quant to count the number of pre-mRNA per nuclei^62^, using populations of 400–500 cells per experiment.

To obtain the number of nascent pre-mRNA per cell, the same images were used without SIM-reconstruction and were analysed using FISH-quant. Briefly, TS were first identified manually, and isolated pre-mRNA molecules located in the nucleoplasm were used to define the PSF and the total light intensity of single molecules, which finally allowed to determine the intensity of TS.

### Live-cell image acquisition

Cells were plated on 25-mm diameter coverslips (0.17-mm thick) in non-fluorescent media^61^. Coverslips were mounted in a temperature-controlled chamber with CO_2_ and imaged on an inverted OMX Deltavision microscope in time-lapse mode. A × 100, NA 1.4 objective was used, with an intermediate × 2 lens and an Evolve 512 × 512 EMCCD camera (Photometrics). Stacks of 11 planes with a *z*-spacing of 0.6 μm were acquired. This spacing still allowed accurate PSF determination without excessive oversampling. Illuminating light and exposure time were set to the lowest values that still allowed visualization of single molecules of pre-mRNAs (laser at 1% of full power, exposure of 15 ms per plane). This minimizes bleaching and maximizes the number of frames that can be collected. Yet, it guarantees that transcription can be detected early on, when one or a few nascent chains are in the process of being transcribed. For short movies, one stack was recorded every 3 s for 15 min. Movies recorded at one stack per second yielded similar parameter values for convoy, but this rapid acquisition reduced movie length, making it difficult to obtain enough data for statistical analyses. For long movies, one stack was recorded every three minutes for 8 h. Movies were analysed with dedicated software. All software tools are available upon request (MS2-quant, RampFinder, RampFitter, ON-quant; see Supplementary Notes 1-3).

### Analysis of short movies and single-molecule calibration

To extract the TS signal in the movies, we manually defined the nuclear outline and the region within which the TS is visible. The stack was corrected for photobleaching by measuring the fluorescence loss of the entire nucleus and fitting this curve with a sum of three exponentials. This fitted curve was then used to renormalize each time point such that its nuclear intensity was equal to the intensity of the first time point. We then filtered the image with a two-state Gaussian filtre^62^. First, the image was convolved with a larger kernel to obtain a background image, which was then subtracted from the original image before the quantification is performed. Second, the background-subtracted image was smoothened with a smaller Kernel, which enhances the SNR of single particles to facilitate spot pre-detection^63^.

We then pre-detected the position of the TS in each frame of the filtered image by determining in the user-specified region the brightest pixel above a user-defined threshold. If no pixel was above the threshold, the last known TS position was used. Pre-detected position was manually inspected and corrected. Then the TS signal was fitted with a 3D Gaussian estimating its s.d. *σ*_*xz*_ and *σ*_*z*_, amplitude, background and position. We performed two rounds of fitting: in the first round all fitting parameters were unconstrained. In the second round, the allowed range was restricted for some parameters, to reduce large fluctuations in the estimates, especially for the frames with a dim or no detectable TS. More specifically, the *σ*_*xz*_ and *σ*_*z*_ were restricted to the estimated median value±s.d. from the frames where the TS could be pre-detected, and the background was restricted to the median value. The TS intensity was finally quantified by estimating the integrated intensity above background expressed in arbitrary intensity units.

With the live-cell acquisition settings, the illumination power was low and we could not reliably detect all individual molecules. We therefore collected right after the end of the movie one 3D stack—termed calibration stack—with increased laser intensity (50% of max intensity, compared with 1% for the movie), which allowed reliable detection of individual RNA molecules (see for example, Fig. 1c). We also collected slices with a smaller *z*-spacing for a better quantification accuracy (21 slices every 300 nm). We found that for our MS2 system the single pre-mRNA intensities correlated with the average MCP-GFP brightness of the cell, that is, the available MCP-GFP (Supplementary Fig. 1H). This required to adjust the quantification workflow of FISH-quant as follows: (a) when calculating the averaged image of single RNA molecules, we subtracted the estimated background from each cell to minimize the impact of the different backgrounds; (b) when quantifying the TS in a given cell, we rescaled the average image of single RNA molecules such that it had the same integrated intensity as the molecules detected in the analysed cell.

To calibrate the MS2 data, that is, to express the measured TS intensity as a function of nascent transcripts, we used the fact that the last movie frame was acquired at the same time as the calibration stack. We then normalized the extracted MS2 data *I*_MS2_ to get the nascent counts *N*_nasc;calib_:


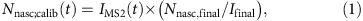


where *N*_nasc,final_ stands for the estimated number of nascent transcripts in the calibration stack and *I*_final_ for the averaged intensity of the last four frames. Note that the approach was limited to movies where the TS was active at the movie end since otherwise its intensity cannot be quantified.

To assess the reliability of the quantification scheme, we looked at the data where we knew how many transcripts we should detect—the nucleoplasmic, released pre-mRNA data. Here we should estimate on average 1 transcript per pre-mRNA molecule. We validated the quantification in two different experiments. First, we looked at the pre-mRNA data obtained from movies where illumination was set to visualize single pre-mRNAs during all movie frames. Using our quantification scheme, we estimated values of 1.05±0.3. To further validate the calibration approach, we compared the estimated nascent mRNA signal from smFISH data and MS2 data. For this purpose, we performed smFISH with probes against the MS2 repeat and quantified the TS signal in >400 cells. We then analysed movies with the described MS2 calibration approach and determined the nascent counts throughout all movies. The obtained mean/median value and s.d. are very close, yielding and additional validation (Supplementary Figure 1I).

The image analysis software is freely available on bitbucket https://bitbucket.org/muellerflorian/ms2_quant, together with examples of the processed data. The software for fitting the live-cell TS intensity with the model of polymerase convoy will be provided on specific request to E. Bertrand. The entire raw dataset of the live-cell experiments can also be accessed after a specific request to E. Bertrand.

### Analysis of long movies

To quantify the long movies acquired at low frames rate (one 3D stack per 3 min), we developed ON-quant, a rapid analysis tool that identified the ON and OFF periods and measured their length. This did not require an absolute quantification of the number of nascent pre-mRNAs and we therefore defined an intensity threshold, based on the mean intensity of single molecules, under which a TS is considered to be silent, and above which a TS is considered to be active. Before analysis we further smoothed the data with a moving average (window size=3). Last, TS were considered to be ON or OFF only if they were in the respective state for at least two consecutive frames.

### Simulations of cell-to-cell variation in gene expression

Monte-Carlo simulation of promoter states, RNA synthesis and degradation were implemented in R, using models containing one, two or three OFF states. Results are described for a model with two OFF states as schematized in Fig. 5a. Note that a linear model (OFF2<−>OFF1<−>ON) is mathematically equivalent to a branched model (OFF2<−>ON<−>OFF1; see Supplementary Note 6). Step-wise assembly of the PIC, however, argues that a linear model is more relevant to transcription initiation and was thus used. The first state of this model is an active, ON state where polymerase convoys form. A second state is a long OFF state that may arises upon dissociation of TBP, and termed OFF_2_. We distinguished two sub-states, OFF_2a_ and OFF_2b_, to account for the two lifetimes of the long non-permissive periods (9 and 34 min for the 4G mutant). A third state is an intermediate OFF_1_ state that contains TBP but that requires other components to switch to the ON state. Switching between OFF_1_ and ON produces polymerase convoys, with rates *k*_on1_ and *k*_off1_. Switching between OFF_1_ and OFF_2_ states creates alternative permissive/non-permissive periods, with rates *k*_on2_, *k*_off2a_ and *k*_off2b_. The parameter *f1* is the fraction of cells in OFF2a versus total OFF2 states.

To produce a quantitative version of the model, we fixed the values for the combined rate of splicing and degradation of the pre-mRNA intron (*k*_deg_) to 1/75 min^−1^, in agreement with the half-life measured by northern blot (Supplementary Fig. 1C). The rate of release of the nascent pre-RNA from the TS was calculated from smFISH quantifications, using a steady-state approximation with the equation *k*_release_/*k*_deg_=meanMat/meanNasc. MeanMat is the average number of released pre-mRNA per cell, and meanNasc is the average number of nascent RNA per cell (shown in Supplementary Fig. 2). The other rates constants were fixed within plausible ranges compatible with live-cell measurements. The rate *k*_on2a_ was fixed (1/*k*_on2a_=5 min), and for the other parameters, the range of values tested were: 1/*k*_ini_ (2.3; 2.6; 3.0; 3.4; 3.7; 4.0; 4.3; 4.6 s); *f1* (0.6; 0.7; 0.8; 0.9); 1/*k*_on2b_ (20; 30; 40; 50 min); 1/*k*_off1_ (1/90; 1/110; 1/130; 1/160 s); 1/*k*_on1_ (1; 1.5; 2; 2.5; 3 min).Values for *k*_off2_ were calculated to match the mean number of pre-mRNA per cell using steady-state approximation, with the following equation (negative values were not allowed):


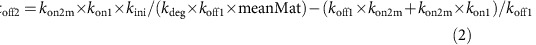


with


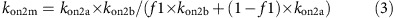


Monte-Carlo simulations were performed for a set of 3,000 cells, using 36,000 iterations of 1-s time-intervals for a total time of 10 h. The resulting distributions of nascent and released pre-mRNAs were then compared with the experimental distributions using *χ*^2^- and KS tests. We used a KS test to compare the distributions of the released, nucleoplasmic pre-mRNAs. The advantage is that KS is independent of binning, and in contrast to *χ*^2^-tests, KS is not limited to the assessment of sampling variability but can also take into account other sources of noise such as technical variations (note, however, that similar results were obtained with KS and *χ*^2^-tests). We used *χ*^2^-tests for nascent RNA because these distributions contained many equal values (for example, zeros, corresponding to silent TS), and this precludes the use of a KS test.

To evaluate the best-fit parameters, *P* values for the distribution of nascent and released pre-mRNAs were multiplied by each other and ranked from the highest to the lowest for all the parameter sets. The 10 best-fit parameters were then averaged. To evaluate experimental reproducibility, the simulations made for one replicate of the experimental distributions were compared with an independent replicate of the experimental distribution. The averaged parameters of the 10 best-fit simulations were similar for both replicates (Supplementary Fig. 6).

### Data availability

The data that support the findings of this study are available from the corresponding authors on request.

## Additional information

**How to cite this article:** Tantale, K. *et al.* A single-molecule view of transcription reveals convoys of RNA polymerases and multi-scale bursting. *Nat. Commun.* 7:12248 doi: 10.1038/ncomms12248 (2016).

## Supplementary Material

Supplementary Information Supplementary Figures 1-7, Supplementary Tables 1-2, Supplementary Notes 1-6 and Supplementary References

Supplementary Movie 1 An isolated transcription cycle in a HeLa cell expressing the WT HIV-1 reporter. Total time is 15 min, frame rate is one 3D stacks per 3 seconds. The left panel shows a high contrast version of the right panel, to display single RNA molecules.

Supplementary Movie 2 An isolated transcription cycle in a HeLa cell expressing the 4G mutant HIV-1 reporter. Total time is 15 min, frame rate is one 3D stacks per 3 seconds. The left panel shows a high contrast version of the right panel, to display single RNA molecules.

Supplementary Movie 3 Cells expressing the WT HIV-1 reporter and recorded for 8 hours at a rate of one 3D image stack every three minutes. The left panel shows a high contrast version of the right panel, to display single RNA molecules. Non-permissive periods are infrequent in WT cells and not visible in this example.

Supplementary Movie 4 Cells expressing the 4G HIV-1 reporter and recorded for 8 hours at a rate of one 3D image stack every three minutes. The left panel shows a high contrast version of the right panel, to display single RNA molecules.
