# Supplementary material for: A single-molecule view of transcription reveals convoys of RNA polymerases and multi-scale bursting
Source: Nat Commun. 2016 Jul 27;7:12248. doi: 10.1038/ncomms12248 (PMC4974459; doi:10.1038/ncomms12248)
Supplement: Supplementary Information — Supplementary Figures 1-7, Supplementary Tables 1-2, Supplementary Notes 1-6 and Supplementary References [file ncomms12248-s1.pdf]

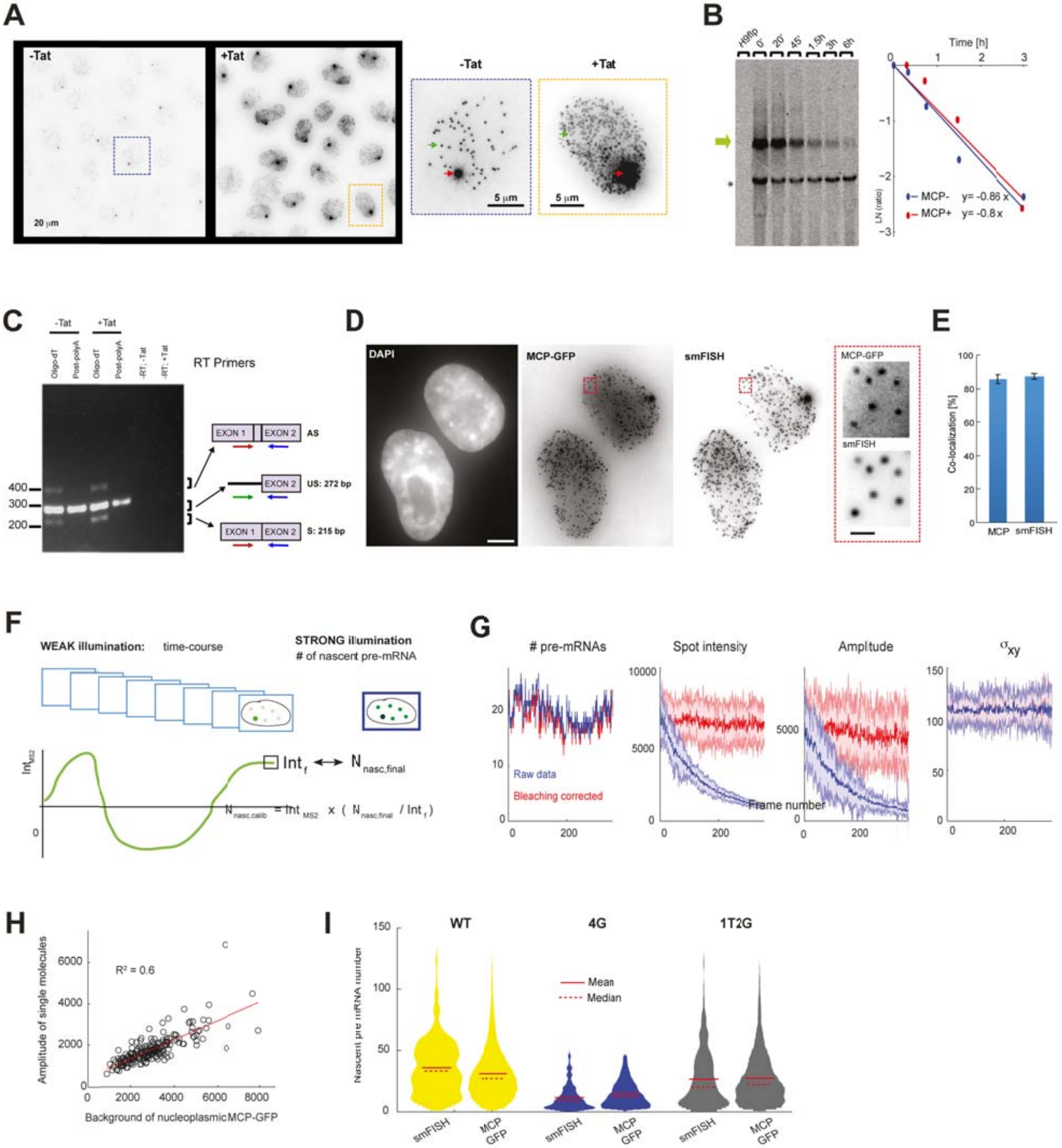

Supplementary Figure 1. Characterization of the HIV-1 reporter tagged with MS2x128

A-Tat-dependent expression of the HIV-1 reporter tagged with the MS2x128 repeat. Panels show HeLa cells in which the HIV-1 pre-mRNA is detected by smFISH. Cells bear a single copy of the

reporter gene integrated with the Flp-In system. Contrast adjustment was identical for the two pairs of images. Right image pair: zoom over one nucleus as indicated by the dashed lines. Left image in each pair: the viral transactivator Tat is not expressed; right image in each pair: Tat is stably expressed. Red arrow: TS; green arrow: single pre-mRNA molecule.

B-Rates of splicing of the HIV-1 pre-mRNA tagged with MS2x128. Left: autoradiogram of a Northern blot probed with the MS2 sequence. Green arrow: unspliced HIV-1 pre-mRNAs. Star: non-specific cross-hybridization of the probe with the transiently transfected Tat plasmid DNA (RNAs were not DNase treated). HeLa Flp-In: untransfected parental cells that do not express the HIV-1 reporter; other lanes correspond to HeLa Flp-In cells expressing Tat and the reporter RNA, and treated with Actinomycin D for the indicated times. Right: curve shows the decay of the HIV-1 pre-mRNA after transcription inhibition, quantified from Northern blots. The pre-mRNA signal was normalized to that of 18S ribosomal RNA, normalized to untreated cells and plotted on a Log scale. Blue curve: HeLa Flp-In cells expressing the reporter and Tat; red curve: cells additionally expressing MCP-GFP. The experiment was repeated four times for cells not expressing MCP-GFP, yielding a pre-mRNA half-life of 45 minutes (standard deviation: 5 minutes).

C-Post-transcriptional splicing of the HIV-1 reporter tagged with MS2x128. Gel shows the result of a competitive PCR assay with three primers (colored in blue, red and green), to analyze the splicing status of HIV-1 MS2x128 reporter, expressed from HeLa Flp-In cells. OligodT: the reverse transcriptase was primed with oligodT to analyze released, nucleoplasmic HIV-1 RNAs; post-polyA: reverse transcriptase priming was done with an oligo that hybridizes downstream the HIV-1 polyA site to analyze 3'-end unprocessed, TS-associated RNAs; -RT, controls without reverse transcriptase. Left bars: molecular weight marker (in bp). S: spliced mRNAs; US: unspliced pre-mRNAs; AS: alternative splicing event occurring 250 nucleotides upstream of the

regular splice acceptor site (but 3' to the MS2 repeat). The PCR reactions were run to completion to compare the patterns obtained in –Tat and +Tat cells independently of the initial amount of cDNA.

D-Detection efficiency of single pre-mRNA molecules with the MS2x128 tag. Images are maximum intensity projections of 3D super-resolved structured-illumination (SIM) images, from HeLa Flp-In cells expressing Tat, MCP-GFP and the HIV-1 reporter tagged with MS2x128. Left panel: nuclei stained with DAPI; middle panel: signal of MCP-GFP; right panel: smFISH performed with probes against the MS2 tag (40 oligonucleotides per RNA molecule). Zoomed right panels: single pre-mRNA molecules are detected by both smFISH and MCP-GFP. Scale bar: 5 μm. Zoom: scale bar is 1 μm.

E-Barplot displaying the percentage of pre-mRNA molecules detected with both MCP-GFP and smFISH. Left bar: the percentage of pre-mRNA molecules detected by smFISH that are also detected with the MCP-GFP; right bar: the opposite quantification. The numbers are from 6 fields of view, representing more than 8000 single pre-mRNA molecules in each channel (18 cells).

F-Schematic illustrating the calibration pipeline. Top: a time-lapse movie is acquired at low illumination power and a calibration stack is acquired at high illumination power at the end of the movie. Bottom: the temporal fluctuations of TS are measured in arbitrary units of fluorescence intensity. The high quality of the calibration stack allows measuring the number of nascent transcripts attached to the TS. The last data point of the movie serves as a reference for renormalization using the estimated nascent mRNA level in the high illumination stack, and assuming that the last movie stack and the calibration stack have TSs with similar intensity (see Methods).

G-Efficiency of photobleaching correction. Individual mRNA molecules were detected in a time-series experiment. Left: graph displays the number of single pre-mRNA detected before (blue) and after (red) bleaching correction. Middle left: total intensities of single pre-mRNA molecules before (blue) and after (red) photobleaching correction. Middle right and right panels: estimated parameters of the 3D Gaussians fitted to single RNA molecules, for the different time-points and without (blue), or with bleaching correction (red).

H-Comparison of the intensity of single RNAs in cells expressing variable levels of MCP-GFP. Each data point summarizes the results of the 3D Gaussian fit applied to all spots detected in one cell (199 calibration stacks were used). The median of the estimated amplitude (y-axis) is plotted as a function of the median of the estimated background of nucleoplasmic MCP-GFP (x-axis). Red line shows a linear fit ($R^2 = 0.6$).

I-Accuracy of live cell calibration. Violin plots show comparison of quantification of active TS with smFISH (left plot for each reporter; N>500), and calibrated value of TS from all time points of MS2 movies (right plot for each reporter; N>60000). Reporters with WT or mutant TATA boxes were used (1T2G and 4G, see Figure 3). Calibrated MS2 values larger than the maximum value obtained by smFISH were removed from the analysis.

A

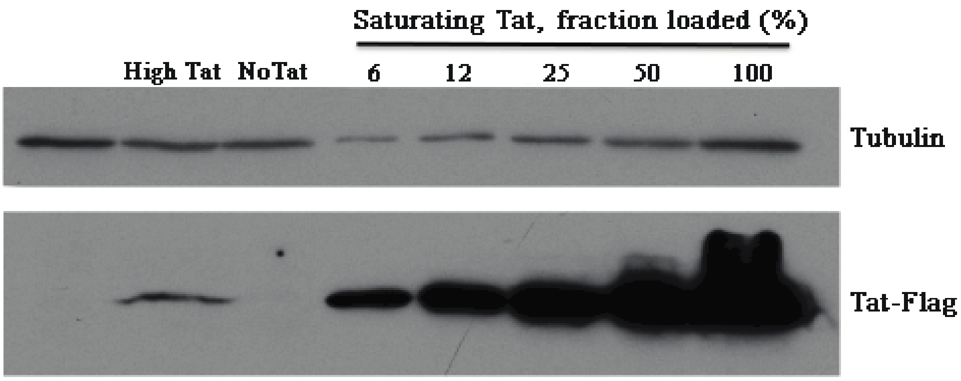

B

Cell line	Nascent pre-mRNA (per cell)	Nucleoplasmic pre-RNA (per cell)	Number of cells
Saturating Tat	38 (+/- 34)	708 (+/- 362)	387
High Tat	32 (+/- 25)	538 (+/- 293)	851

Supplementary Figure 2. Tat levels are saturating in *high Tat* cells

A-Western blot analyses of the Tat expressing cell lines. The antibodies used are indicated on the right. *No Tat*: cells not expressing Tat; *High Tat*: cells used throughout the study; *Saturating Tat*: cells expressing Tat from the chicken β-actin promoter inserted at the AAVS1 locus; various dilution of the extract were loaded, with 100 % corresponding to the amount loaded in the High Tat and No Tat lanes.

B-Levels of nascent and mature HIV-1 pre-mRNAs in the indicated cell lines.

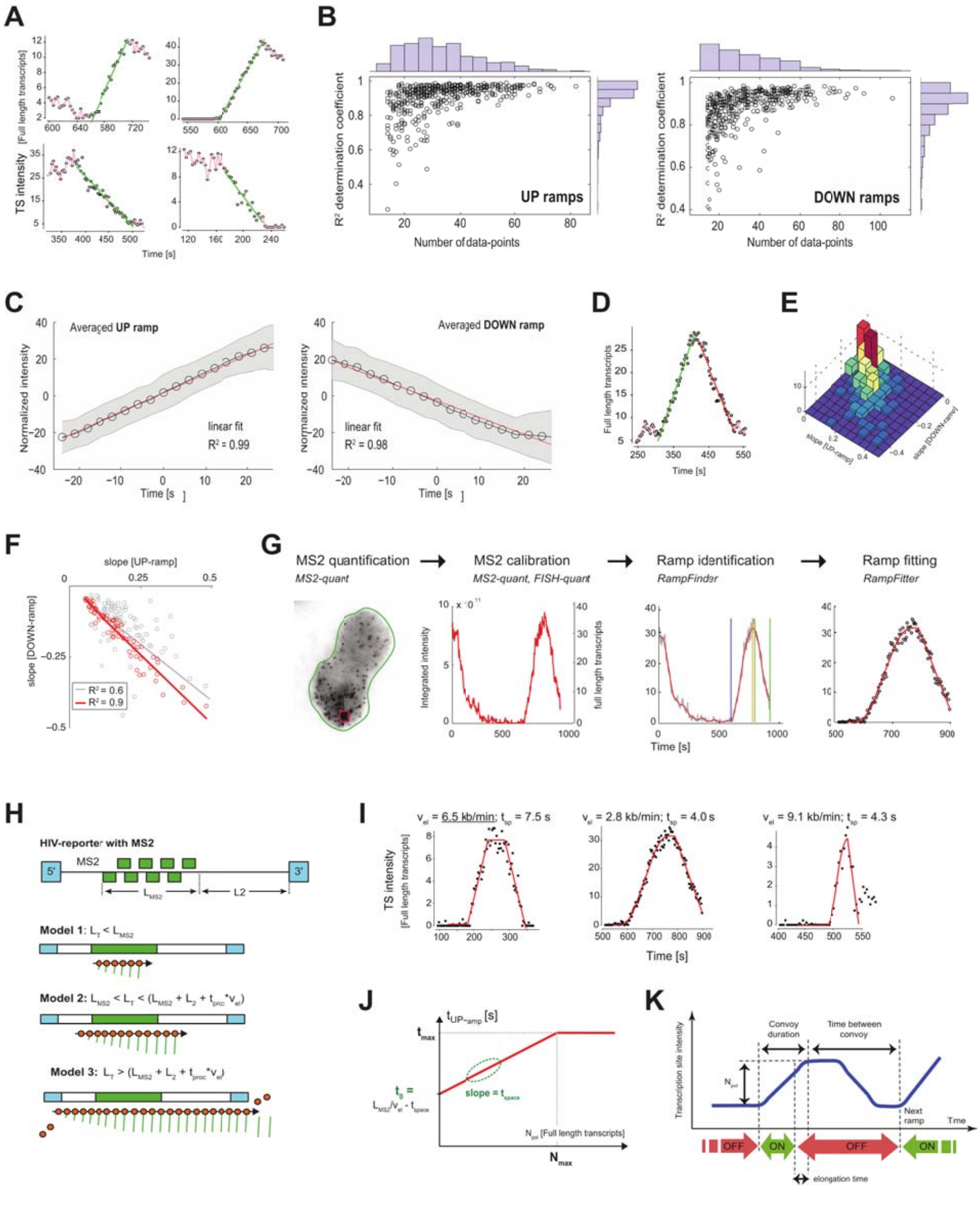
A
B
TS intensity [Full length transcripts]
Time [s]
R² determination coefficient
UP ramps
DOWN ramps
Number of data-points
C
Averaged UP ramp
Averaged DOWN ramp
Normalized intensity
linear fit
R² = 0.99
R² = 0.98
Time [s]
D
Full length transcripts
E
slope [UP-ramp]
slope [DOWN-ramp]
F
R² = 0.6
R² = 0.9
G
MS2 quantification
MS2-quant
MS2 calibration
MS2-quant, FISH-quant
Ramp identification
RampFinder
Ramp fitting
RampFitter
Integrated intensity
full length transcripts
H
HIV-reporter with MS2
MS2
5'
3'
L2
I
TS intensity [Full length transcripts]
Time [s]
J
slope = t_space
[Full length transcripts]
K
Transcription site intensity
Convoy duration
Time between convoy
Next ramp
OFF
ON
elongation time

Supplementary Figure 3. Linearity of UP and DOWN ramps and modeling of polymerase convoys

A-Examples of linear fits of UP (top panels) and DOWN (bottom panels) ramps. Curves display TS intensities expressed in number of pre-mRNA molecules, and plotted as a function of time, in seconds. Pink: plateau before and after a ramp identified with *RampFinder*.

B-Linearity of UP and DOWN ramps. Graph shows the R^2 determination coefficient of linear fits to UP (left) and DOWN (right) ramps, as a function of the number of time-points in the ramp (n>400). Only ramps with a preceding plateau of less than 6 pre-mRNAs were considered. Ramps with less than 10 time points were excluded from the analysis. Each curve is from a single cell recorded for 15 minutes with a frame-rate of one image stack every 3 seconds.

C-Averaged UP and DOWN ramps. Individual ramps of isolated transcription cycles were aligned with their center, normalized with their estimated slopes and averaged (n>400). Red: a linear trend line with its determination coefficient R^2.

D-Example of linear fits to UP and DOWN ramps belonging to the same transcription cycle. Green: UP ramp; Red: DOWN ramp. Note the similar but inverse slopes for the UP and DOWN ramps.

E and F-Correlation between the slopes estimated for UP and DOWN ramps within isolated transcription cycles. E: 2D histogram of the UP and DOWN ramps. F: plot showing the slope of the DOWN ramps as a function of the slope of the UP ramps. Grey: data from all isolated transcription cycles with a similar number of time-points in the UP and DOWN ramp. Red: data from the top the 50% of transcription cycles with the highest correlation between UP and DOWN ramps. This selection is shown because UP and DOWN ramps are expected to be correlated for only a subset of polymerase convoys, where the length of the convoy is longer than the MS2

repeat (see Supplementary Note 1). A linear trend to the data is shown with its determination coefficient.

G-Quantification scheme for polymerase convoys. First (left panel), TS are identified (red square) and quantified using MS2-quant; second (middle left panel), intensities are converted into a number of molecule using *FISH-quant* and *MS2-quant*; third (middle right panel), UP and DOWN ramps are identified with *RampFinder*; fourth (right panel), isolated transcription cycles are fitted to the model of polymerase convoys using *RampFitter* (see Supplementary Note 1-3).

H-Model of polymerase convoys. Top: schematic of the reporter. Bottom: three cases of polymerase convoys that are described with different sets of equations (Supplementary Note 3). N_{pol} : number of polymerase; t_{space} : RNAPII spacing (in seconds); v_{el} : elongation rate; L_T : convoy length (in nucleotides).; L_{MS2}: length of the MS2 repeat; L_2: distance between the end of the MS2 repeat and the polyA signal.

I-Fits of isolated transcription cycles with the polymerase convoy model. Black dots display experimental values of TS intensities (in number of full-length pre-mRNA molecules). Red curves show best model fit. v_{el}: estimated elongation rate; t_{sp}: spacing. Underlined numbers: only a minimum value was estimated (see Supplementary Note 3).

J-Estimation of mean values of t_{space} and v_{el} from population analysis. Graphs displays the duration of UP ramps ($t_{UP\text{-}ramp}$) as a function of N_{pol} (red). N_{max} is the maximum number of polymerases that can be loaded on the gene*;* t_{max} is the maximum possible duration of the UP ramps; t_0 is the intersect at the origin.

K-Measurement of convoy duration and time between convoys from short movies at high temporal resolution (3 seconds). Schematic displaying the various elements of an isolated transcription cycle followed by an UP ramp, and the boundaries used to estimate convoy duration and time between convoys. Note that the time to elongate through the MS2 repeat has to be added

to the time between convoys to obtain an exact value of the promoter OFF time. Likewise, the elongation time should be subtracted from the convoy duration to compute the exact promoter ON time.

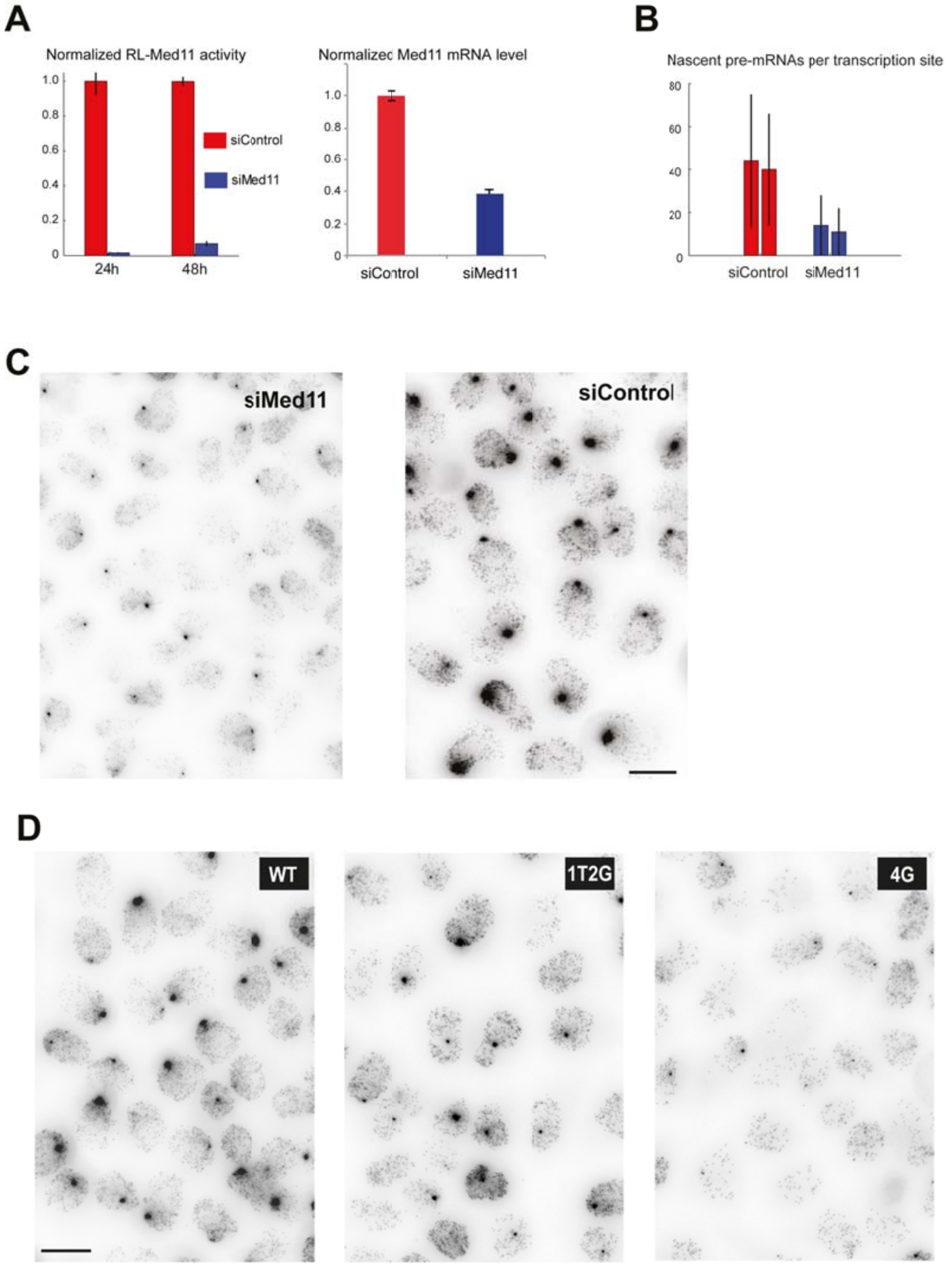

Supplementary Figure 4. Effect of Mediator and TATA box on HIV-1 promoter activity

A-Efficiency of the knock-down of MED11. Left barplot shows normalized Renilla luciferase activity of cells transfected with a MED11-Renilla luciferase construct, a control Firefly-luciferase vector, and either control siRNAs (Control) or siRNA targeting MED11 (siMED11).

Renilla luciferase activity was normalized to that of Firefly luciferase and control siRNA was set to 1. Right barplot shows MED11 mRNA levels as measured by RT-qPCR, after treatment of cells with either a control or a MED11 siRNA for 24h. RNA levels were normalized to that of GAPDH mRNA.

B-Barplot shows the decrease in nascent HIV-1 RNA expression, in cells treated with the MED11 siRNA and control siRNA. Expression levels were estimated by single molecule counting from smFISH images, using FISH-quant (see Methods).

C-Effect of siRNA against MED11 on HIV-1 reporter gene expression. Maximum intensity projections of smFISH images of *High Tat* cells expressing the HIV-1 reporter gene with MS2x128 tag and treated with control siRNAs (Control) or siRNAs targeting MED11 (siMED11). Cells expressed Tat and MCP-GFP. Image contrast adjustment was identical for all images. Scale bar: 20 µm.

D-Expression of the tagged HIV-1 pre-mRNA from WT and TATA-box promoter mutants in Hela cells. Images are maximum intensity projections of smFISH images of cells expressing the indicated reporter. Contrast adjustment was identical for all images. TSs are saturated because of the rescaling required to visualize single molecules. Scale bar: 10 µm.

A

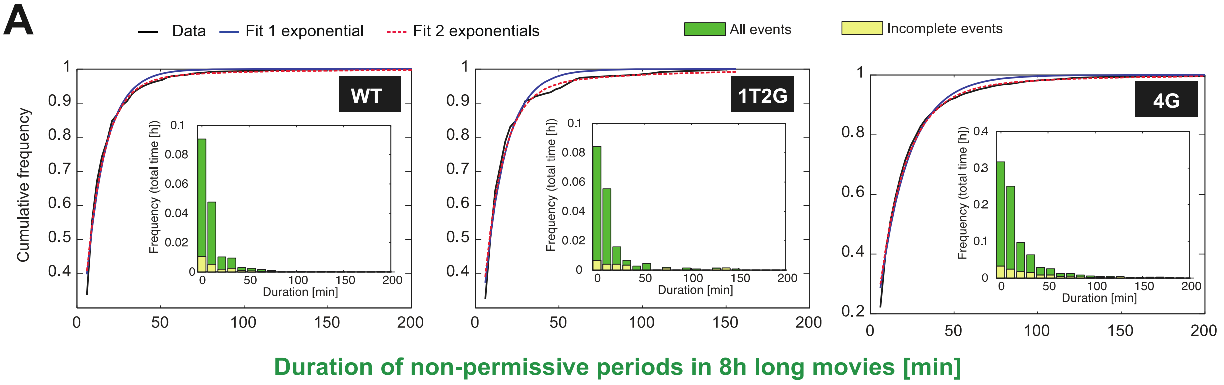

B

2 exponential equation :

$$cdf = 1 - f_1 * \exp(-t/t_1) - (1-f_1) * \exp(-t/t_2)$$

fitted values	f_1	t_1 [min]	t_2 [min]
WT	0.79	5.0	19.7
1T2G	0.87	6.5	34.0
4G	0.77	9.2	34.1

Duration of non-permissive periods in 8h long movies [min]

C

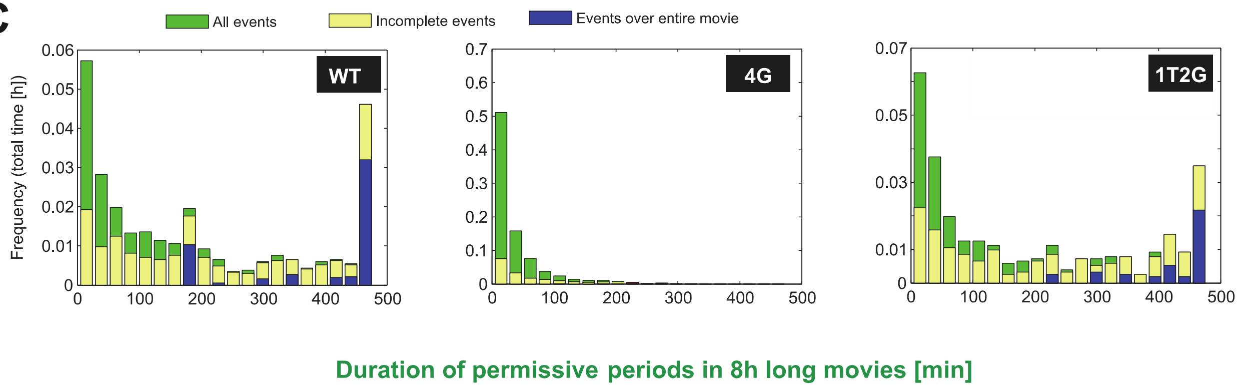

Supplementary Figure 5. Distributions of permissive and non-permissive periods in WT and TATA mutants HIV-1 reporters

A-Cumulative distribution of non-permissive periods in the 8h movies (black) and mono-exponential (blue) and bi-exponential (red) fits, for the WT and the 1T2G and 4G reporters. Insets, frequencies (h^{-1}).

B-Best fitting parameters of the cumulative distribution frequencies of the non-permissive period duration in the 8h movies. Cdf: cumulative density function.

C-Distributions of permissive periods in the 8h movies, for the WT, 1T2G and 4G reporters. Permissive periods are not expected to follow an exponential distribution because the pre-mRNA remains at the TS for about two minutes after its synthesis, after the promoter turns OFF. The promoter can thus switch back ON before releasing all its pre-mRNAs. Several individual ON periods can thus be merged and considered as a single event, resulting in complex distributions for the duration of permissive periods. This is especially visible for the WT and 1T2G reporters, because of their low frequencies of non-permissive periods (see panel A, insets, and Figure 4).

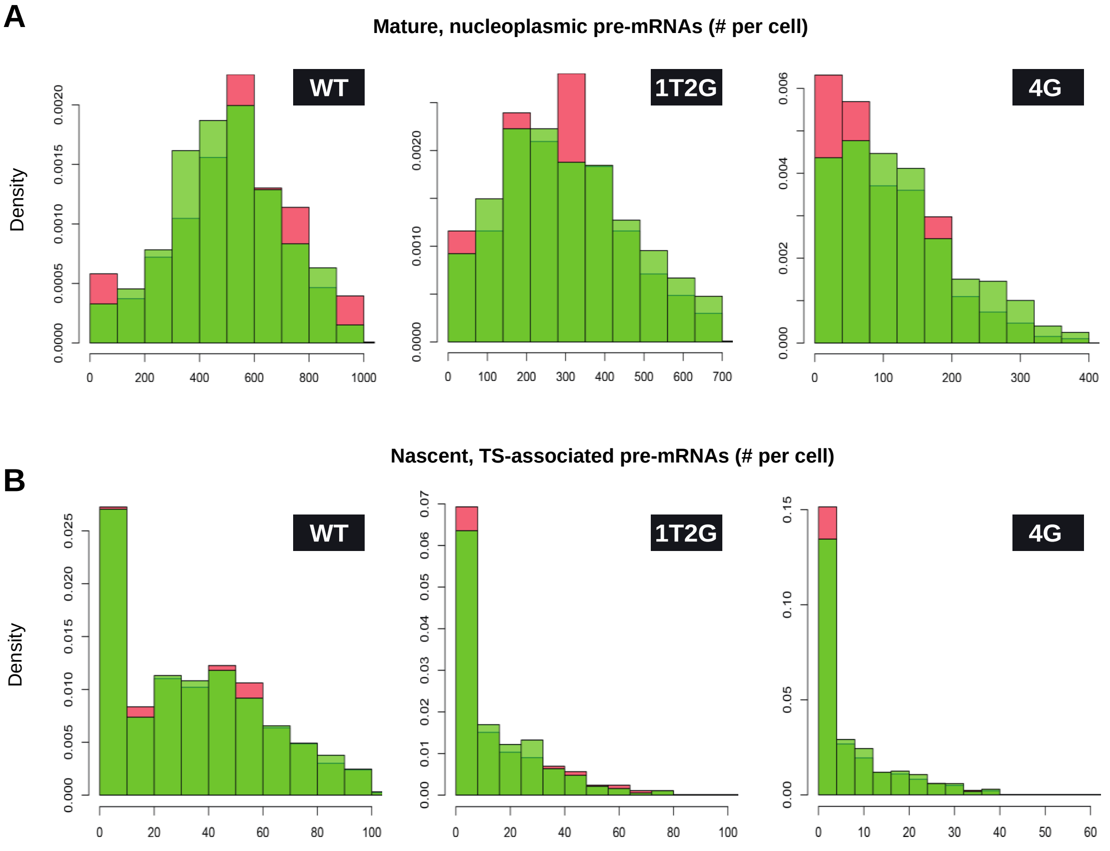

C

1/rate [min]	k_{on1a}	k_{on1b}	f1	k_{off1}	k_{on2}	k_{off2}	k_{ini} [s]
WT-rep1	5	33.4 (6.7)	0.69 (0.11)	11.3 (6.8)	2.0 (0.5)	2.1 (0.5)	2.75 (0.2)
WT-rep2	5	36.0 (8.4)	0.64 (0.08)	13.6 (5.9)	2.55 (0.4)	2.1 (0.4)	2.39 (0.3)
1T2G-rep1	5	32.0 (6.3)	0.6 (0.02)	8.1 (1.9)	2.75 (0.4)	1.6 (0.2)	2.51 (0.2)
1T2G-rep2	5	49.0 (3.1)	0.64 (0.08)	10.0 (3.7)	2.65 (0.4)	1.9 (0.5)	2.7 (0.2)
4G-rep1	5	50 (0.0)	0.72 (0.10)	5.4 (2.2)	2.5 (0.6)	1.6 (0.1)	4.3 (0.2)
4G-rep2	5	49 (3.2)	0.67 (0.09)	4.2 (1.9)	2.55 (0.6)	1.9 (0.4)	3.9 (0.4)
Live	6.9 (2.5)	29.2 (8.2)	0.81 (0.06)	n. d.	2.4 (1.5)	1.5 (0.5)	4.1 (2.5)

Supplementary Figure 6. Replicate analysis of HIV-1 smFISH in *High Tat* cells and parameter values for distribution fits

A-B-Comparison of independent replicates of smFISH data. Histograms show the distribution of the number of released pre-mRNA per cell in the cell population, for each of the indicated

reporter (> 400 cells for each replicate). Red: replicate 1; green replicate 2. SmFISH was performed with a set of 40 probes against the MS2 repeat and images were acquired on an OMX microscope using SIM-reconstruction to obtain super-resolution images. Released (A) and nascent (B) pre-mRNAs were counted with FISH-quant.

C-Comparison of the parameter values of the best fitting simulations for the two replicates of the experimental distributions. Values are averages of the 10 best-fitting simulations using the model of Figure 4A; standard deviation in parenthesis. Underlined: the value for k_{on2a} was fixed (see Methods).

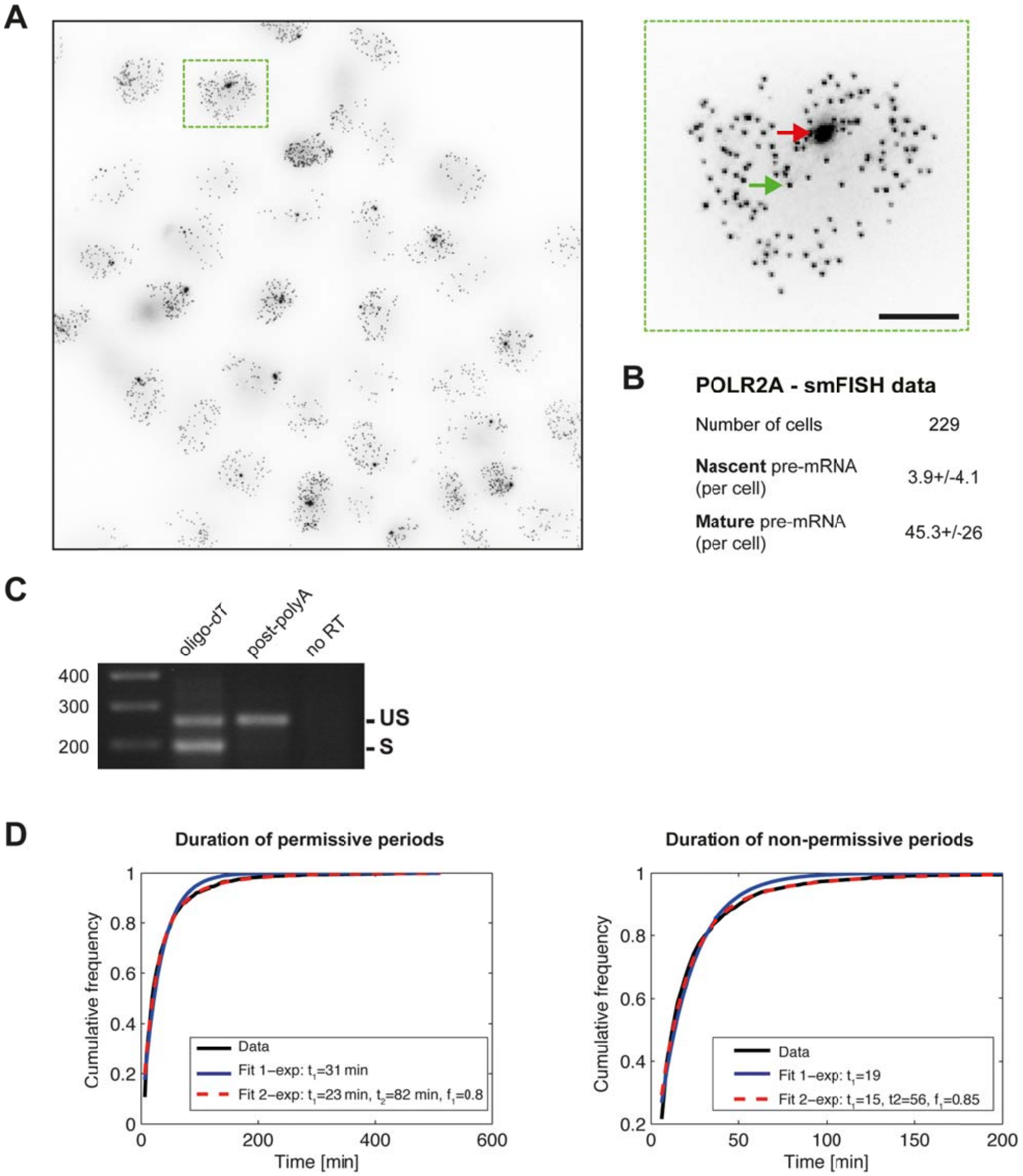

Supplementary Figure 7. Characterization of POLR2A-MS2x128 reporter cells

A-Expression of the MS2x128 tagged POLR2A pre-mRNAs in Hela reporter cells. Images are maximum intensity projections of smFISH images of cells expressing the POLR2A-MS2x128 reporter. TSs are saturated because of the rescaling to visualize single molecules. Scale bar: 5 µm.

B- Levels of nascent and mature POLR2A-MS2x128 pre-mRNAs in the reporter cell line.

C-Post-transcriptional splicing of the Pol-II-1 reporter tagged with MS2x128. Gel shows the result of a competitive PCR assay to analyze the splicing status of Pol-II MS2x128 reporter, expressed from HeLa Flp-In cells. Three primers were used as in Supplementary Figure 1C, except that the intronic primer was located on the other side of the intron, explained why the AS species was not detected (see Supplementary Figure 1C). OligodT: the reverse transcriptase was primed with oligodT to analyze released, nucleoplasmic Pol-II MS2x128 RNAs; post-polyA: reverse transcriptase priming was done with an oligo that hybridizes downstream the HIV-1 polyA site to analyze 3'-end unprocessed, TS-associated RNAs; -RT, controls without reverse transcriptase. Left bars: molecular weight marker (in bp). S: spliced mRNAs; US: unspliced pre-mRNAs.

D-Cumulative distribution of permissive and non-permissive periods in the long movies (black) and mono-exponential (blue) and bi-exponential (red) fits, for the POLR2A reporter.

Supplementary Table I. Comparison of simulated and experimental polymerase trains

Simulation parameters t_{space} (s); v_{el} (kb/min); t_{proc} (s)	t_{space} (STD)	v_{el} (STD)	t_{proc} (STD)	Residual STD
t_{space} =2; v_{el} =4, t_{proc} =100; expo	2.0 s (0.5)	4.2 (0.8)	102 s (9)	1.6
t_{space} =2; v_{el} =8, t_{proc} =100; expo	4.0 s (1.0)	7.8 (2.3)	99 s (17)	1.7
t_{space} =4; v_{el} =4, t_{proc} =100; expo	3.9 s (1.0)	3.7 (1.5)	100 s (27)	1.6
t_{space} =4; v_{el} =4, t_{proc} =100; unif	3.9 s (0.3)	3.8 (1.5)	103 s (21)	1.6
Real data	4.2 s (2.6)	3.7 (2.1)	105 s (60)	1.6

One thousand curves were simulated per condition. t_{space}: spacing between polymerases; v_{el} : elongation rate; t_{proc} : 3'-end processing time; expo: exponential spacing; unif: uniform spacing. In all cases, the number of polymerases in the convoys was 20, the mean number of polymerases found in the HIV-1 convoys.

Supplementary Table II. Comparison of polymerase clusters and convoys.

	Poymerase spacing	Number of polymerases	Distance between convoys or clusters
HIV-1 convoys[1]	280 bases	19	6.8 kb
Typical liver polymerase cluster[2]	750 bases	12.5	n. d.[4]
Atypical liver polymerase cluster[2]	150 bases	60	n. d.
Drosophila embryos[3]	90 bases	n.d.	1-5 kb

[1]data from Figure 2; gaps per convoy were computed by multiplying the time between convoy by the mean elongation rate.

[2]data from reference[1].

[3]data from reference[2].

[4]not determined

Supplementary Note 1: pooled analysis of isolated transcription cycles

Linearity of UP and DOWN ramps indicates key properties of polymerase convoys

The UP and DOWN ramps of many isolated transcriptional cycles appeared to be linear (Supplementary Figure 3A). To quantify the linearity of UP and DOWN ramps, they were fit with linear models. R^2 coefficient and residual normality, evaluated with a Shapiro test, were recorded for each ramp. We observed a median determination coefficient higher than 0.92, with 34 time points per ramp and a normal distribution for residuals (Supplementary Figure 3B, not shown). Linearity was confirmed by averaging UP and DOWN ramps, using a set of 450 isolated transcription cycles, and observed a determination coefficient of 0.98 (Supplementary Figure 3C).

The linearity of the ramps allows inferring important biological properties of polymerase convoys. First, the linearity of DOWN ramps indicates a regular release of pre-mRNAs, which could be explained by regularly spaced polymerases that reached the polyA signal at regular time intervals. Second, a linear UP ramp indicates a constant synthesis rate of MS2 stem-loops, which can be interpreted differently depending on the distance between the first and last polymerase. If this distance is shorter than the MS2 repeat (Supplementary Figure 3H, model 1), then the simplest explanation is an uniform and constant speed for the polymerases. If this distance is larger than the MS2 repeat (Supplementary Figure 3H, model 2), then a linear UP ramp implies that both the number of polymerases on the repeat and their velocities are constant, also indicating that polymerases are regularly spaced and move at uniform speed. In this situation, the model of polymerase convoys further predicts that slopes of the UP and DOWN ramps are identical (see calculation in next section), a feature that is in fact visible in the majority of the isolated transcriptional cycles (Supplementary Figure 3D-F). Of note, we observed these linear ramps not only in isolated cycles but also throughout most of the movies (Figure 2A). Thus, they represent the usual mode of activity of the HIV-1 promoter. The linearity of UP and DOWN

ramps thus indicates that isolated transcription cycles are produced by a set of regularly spaced polymerases that move at the same speed.

The model of polymerase convoy*s predicts linear phases during UP and DOWN ramps*

The UP ramp is observed during the onset of transcription when MS2 stem loops are produced by the polymerase convoy and its slope depends on synthesis rate of the MS2 stem-loops. In most conditions this curve has a linear segment, and several cases should be considered depending on the convoy length (L_T). These cases are refereed to as model 1, 2 or 3 and are depicted in Supplementary Figure 3H.

In the case of Model 1 ($L_T<L_{MS2}$), the synthesis rate of the MS2 stem-loops by a polymerase convoy becomes constant at the moment the last polymerase enters the repeat and until the first polymerase arrives at the repeat end (see schematic in Supplementary Figure 3H). During this time, the UP ramp is linear and its slope is equal to the elongation rate v_{el} multiplied by the number of polymerases on the repeat, i.e. $Npol^* \, v_{el}$ nucleotides per second (or $Npol^* \, v_{el}$ $/L_{MS2}$ molecules of MS2x128 repeat per second).

In the case of Model 2 ($L_{MS2}<L_T<L2+t_{proc}^* \, v_{el}$), the total synthesis rate is constant during the time period that starts after the first polymerase arrives at the end of the MS2 repeat and that ends when the last polymerase enters the repeat (see schematic Supplementary Figure 3H). During this time, the synthesis rate of the MS2 stem-loops is equal to v_{el} multiplied by the number of polymerases on the MS2 repeat ($L_{MS2}/(v_{el} * t_{space})$), i.e. $1/ t_{space}$ pre-mRNA molecules per second. Note that for $L_T=L_{MS2}$, there is no linear phase, but the synthesis rate at the inflexion point is also $1/ t_{space}$.

The case of Model 3 ($L_T>L2+t_{proc}^* \, v_{el} +L_{MS2}$) is identical to Model 2, except that the linear increasing phase ends when the first polymerase is released from the gene.

The DOWN ramps are determined by the release of RNA molecules from the TS. Assuming that the rate of 3'-end processing is constant, RNAs will be released at regular time intervals determined by t_{space}. Under these conditions, the DOWN ramps are linear and have a slope of 1/ t_{space} for all models.

Estimation of mean values of v_{el} and t_{space} from a regression analysis of pooled UP ramps

With a set of UP ramps, the duration of the UP ramps can be used to estimate the mean values of v_{el} and t_{space}. UP ramps start when the first polymerase enters the MS2 repeat and terminates when the plateau or peak intensity is reached. $T_{UP\text{-}ramp}$ depends on the model and can be estimated as:

Model 1 & 2: $T_{UP\text{-}ramp} = L_{MS2}/ v_{el} + (N_{pol}-1) * t_{space}$

Model 3: $T_{UP\text{-}ramp} = (L_{MS2}+L_2)/ v_{el} + t_{proc}$

Given that few or no UP ramps fall in the category of model 3 (less than 1%, see Supplementary File with fit values of all transcription cycles), plotting the UP ramp duration as a function of N_{pol} should yield a straight line with the slope being t_{space} and the intercept at the origin $L_{MS2}/ v_{el} - t_{space}$ (Supplementary Figure 3J). Since N_{pol} and $T_{UP\text{-}ramp}$ can be directly measured from *RampFinder* in absence of any model fitting (see above), averaged values for t_{space} and v_{el} can be directly estimated using a set of UP ramps derived from isolated transcription cycles (Figure 2F). This yields a mean spacing of 4.3 s and an elongation rate of 4.1 kb/min. This in good agreement with the values derived analyzing only DOWN ramps (average value of 4.1s for t_{space}), or from the fits of individual convoys (average values of 4.1s for t_{space} and 4 kb/min for v_{el}).

Supplementary Note 2: automated identification of ramps and isolated transcription cycles: RampFinder

The calibrated time traces measuring the number of RNA molecules of TS over time were analyzed with dedicated tools to identify UP and DOWN ramps, as well as isolated transcription cycles. *RampFinder* was written in R and utilized user-defined criteria to recognize the various elements of the transcription cycles: plateau, UP ramps and DOWN ramps. In practice, raw data were first smoothened using a local polynomial averaging using 20 data points. Plateau were defined by a series of at least 2 points with a mean slope comprised between –0.033 and +0.033 RNA per second and with a maximal slope lower than 0.06. UP-ramps must be preceded by a plateau and should additionally contain a series of at least 6 points with a positive slope greater than 0.06 RNA/s. DOWN-ramps were defined by at least 6 points with a slope lower than -0.06 RNA/s. Manual examination of the results indicated that these parameters allowed reliable identification of ramps. Isolated transcription cycles were finally defined by a succession of either plateau-UP ramp-DOWN ramp, or plateau-UP ramp-plateau-DOWN ramp, with the mean value of the first plateau having less than 6 polymerases. The raw calibrated data corresponding to isolated transcription cycles were then displayed and manually inspected, and finally stored in a file for future use with the fitting routine (*RampFitter*, see below). To measure the duration between two transcription cycles, we also recorded the start of the next UP-ramp.

Supplementary Note 3: modeling individual polymerase convoys and estimation of convoy parameters

RampFitter

To extract quantitative parameters of transcription, isolated transcription cycles were fit with the polymerase convoy model using a dedicated script in R (*RampFitter*). The model predicts the TS intensity as a function of the progression of a polymerase convoy across the reporter gene, and it has four variables (see Figure 2C, Supplementary Figure 3H and below) : N_{pol}, the number of polymerases in the convoy; t_{space}, the time corresponding to the passage of two successive polymerases on the convoy (in seconds); v_{el}, the elongation rate (in nucleotide/second), and t_{proc}, the time required for 3'-end processing and release of the pre-mRNA from the TS (in seconds).

To derive exact solutions of this model, three cases were considered as above, referred to as Model 1, Model 2, and Model 3 (see schematic in Supplementary Figure 3H). *I(t)* is the intensity of the TS (expressed in the number of full length molecules of MS2x128 repeats) over time, L_T the length of the polymerase convoy in nucleotide, L_{MS2} the length of the MS2 repeat, L_2 the length of the fragment separating the MS2 repeat from the polyA site, $N_{pol,ini}$ the signal at the start of the transcription cycle (in number of pre-mRNA molecule), t_0 the time at which the first polymerase enters the MS2 repeat, *t* the time. Depending on the time passed and the length of the polymerase convoy compared to the other elements, different phases, thereafter noted I_i, can be defined. We defined an additional variable to increase the readability of the following equations: $\Delta t = t - t_0$, is the time passed since the first polymerase entered the repeat;

<u>Model 1 (L_T<L_{MS2})</u>

If $L_T < (L_2 + t_{proc} * V_{el})$:

$\Delta t < 0$: *before first polymerase enters repeat*

$I_1(t) = N_{pol,ini}$

$0 < \Delta t \leq L_T/V_{el}$: *after first polymerase entered repeat*

$I_2(t) = \Delta t * \Delta t * V_{el} /(t_{space} *2) + N_{pol,ini}$

$L_T/V_{el} < \Delta t \leq L_{MS2}/V_{el}$: *all polymerases entered the repeat*

$I_3(t) = (\Delta t - L_T/V_{el})*N_{pol}*V_{el} + (L_T/V_{el})*(L_T/V_{el})*V_{el}/(t_{space}*2) + N_{pol,ini}$

$L_{MS2}/V_{el} < \Delta t \leq (L_T + L_{MS2})/V_{el}$: *first polymerase exits the repeat*

$I_4(t) = L*N_{pol} + (L_{MS2} - L)*N_{pol} - ((L_{MS2}+L)/V_{el} - t + t_0)*((L_{MS2}+L_T)/V_{el} - t + t_0)*V_{el}/(t_{space}*2) + N_{pol,ini}$

$(L_T + L_{MS2})/V_{el} < (\Delta t) \leq L_{MS2}/V_{el} + t_{proc} + L_2/V_{el}$: *last polymerase exits the repeat*

$I_5(t) = L_T*N_{pol} + (L_{MS2} - L_T)*N_{pol} + N_{pol,ini}$

$L_{MS2}/V_{el} + t_{proc} + L2/V_{el} < \Delta t$: *polymerases get released*

$I_7(t) = N_{pol} + N_{pol,ini} - (\Delta t + L_{MS2}/V_{el} + t_{proc} + L_2/V_{el})/t_{space}$

If $L_T > (L_2 + t_{proc} * V_{el}))$:

$\Delta t < 0$: $I_1(t)$

$0 < \Delta t \leq L_T/V_{el}$: $I_2(t)$

$L_T/V_{el} < \Delta t \leq L_{MS2}/V_{el}$: $I_3(t)$

$L_{MS2}/V_{el} < \Delta t \leq (L_2 + L_{MS2})/V_{el} + t_{proc}$: $I_4(t)$

$(L_2 + L_{MS2})/V_{el} + t_{proc} < \Delta t \leq L_{MS2}/V_{el} + N_{pol} * t_{space}$: *first polymerase is released*

$I_6(t) = (L_{MS2} * N_{pol} - ((L_{MS2}+L)/V_{el} - t + t_0)*((L_{MS2}+L_T)/V_{el} - t + t_0)*V_{el}/(2*t_{space})) + N_{pol,ini} -$

$(t - (t_0 + L_{MS2}/V_{el} + t_{proc} + L_2/V_{el}))/t_{space}$

$L_{MS2}/V_{el} + N_{pol} * t_{space} < \Delta t$: $I_7(t)$

Model 2 ($L_{MS2} \leq L_T \leq L_2 + t_{proc} * v_{el} + L_{MS2}$)).

If $L_T < (L_2 + t_{proc} * V_{el})$:

$\Delta t < 0$: $I_1(t)$

$0 < \Delta t \leq L_{MS2}/V_{el}$: $I_2(t)$

$L_{MS2}/V_{el} < \Delta t \leq (N_{pol} * V_{el} * t_{space})/V_{el}$: *all polymerases entered the repeat*

$I_8(t) = ((t - L_{MS2}/V_{el} - t_0)*(L_{MS2}/t_{space}) + (L_{MS2}/V_{el})*(L_{MS2})/(2*t_{space})) + N_{pol,ini}$

$(N_{pol} * V_{el} * t_{space}) / V_{el} < \Delta t \leq (N_{pol} * V_{el} * t_{space} + L_{MS2}) / V_{el}$: *first polymerase exits the repeat*

$$I_9(t) = (L_{MS2} * N_{pol} - ((L_{MS2}+L_T)/ V_{el} - t + t_0) * ((L_{MS2}+L)/ V_{el} - t + t_0) * V_{el} /(2 * t_{space})) + N_{pol,ini}$$

$(N_{pol} * V_{el} * t_{space} + L_{MS2}) / V_{el} < \Delta t \leq (L_{MS2}/ V_{el} + t_{proc} + L_2 / V_{el}$: $I_5(t)$

$L_{MS2}/ V_{el} + t_{proc} + L_2/ V_{el} < \Delta t$: $I_7(t)$

If $L_T \geq (L_{MS2}+L_2+t_{proc} * V_{el})$:

$t \leq t_0$: $I_1(t)$

$0 < \Delta t \leq L_{MS2}/ V_{el}$: $I_2(t)$

$L_{MS2}/ V_{el} < \Delta t \leq (N_{pol} * V_{el} * t_{space}) / V_{el}$: $I_8(t)$

$(N_{pol} * V_{el} * t_{space}) / V_{el} < \Delta t \leq L_{MS2}/ V_{el} + t_{proc} + L_2/ V_{el}$: $I_9(t)$

$L_{MS2}/ V_{el} + t_{proc} + L_2/ V_{el} < \Delta t \leq (L_{MS2}/ V_{el} + N_{pol} * t_{space})$: $I_6(t)$

$(L_{MS2}/ V_{el} + N_{pol} * t_{space}) < \Delta t$: *polymerases are released*

$$I_{10}(t) = L_2/(t_{space} * V_{el}) + t_{proc}/ t_{space} + N_{pol,ini} - (t-(t0+ L_{MS2}/ V_{el} + N_{pol} * t_{space})) / t_{space}$$

Model 3: $L_T > L2 + t_{proc} * v_{el} + L_{MS2}$.

Equations for this model were not implemented because only few transcriptional cycles fell in this category (less than 3%, see below). When model 3 was encountered in the fitting routine, a warning was recorded and the data was not fit.

Fitting the function *I(t)* to the data was done using a non-linear minimization routine in R. Model 1 and 2 were included in the function minimized and selected according to convoy length, such that a single solution was obtained. Note that the equations are correct only for $L2 \leq L_{MS2}$.

Confidence intervals and limits in the measurements of v_{el}

In the case of model 2 ($L_T > L_{MS2}$), the slope of the linear part of the UP ramp is $1/ t_{space}$ and v_{el} impacts only the non-linear phase of the UP ramp, i.e. the shoulders before and after the linear

phase. If the convoy is long compared to the MS2 repeat, most of the data points will be in the linear phase and only a small fraction of the data will thus depend on v_{el}. In this case, v_{el} cannot be determined accurately. In practice, this occurred in about 60% of the cases, in which only a minimal value was estimated. This value was determined by systematically decreasing v_{el} starting with the best fit value, and used a t-test to determine if the fit significantly worsens, taking into account the experimental variability.

Fitting the individual transcription cycles indicated that 82% correspond to Model 2, 15% to Model 1, and less than 3% to Model 3. This agreed with the analysis of the slope of the UP and DOWN ramps, which in the majority of the cases were equal as expected from Model 2 (see Supplementary Figure 3D-F). t-statistics were used to evaluate how well the parameters were determined by the fit. t_{space} was well determined in 95% of the cases, t_{proc} in 60% of the cases and v_{el} in only 40% of the cases. In the other 60% of the cases, a minimal v_{el} value was computed as described above.

Simulation and spacing variability

In order to test the ability of our model to correctly estimate the parameters of polymerase convoys, we performed simulations. We simulated polymerases initiating and moving through the HIV-1 reporter gene, and calculated the number of RNA produced as function of time. For each condition (each row in the table below), we simulated 1,000 convoys and added Gaussian noise of 1.6 RNA. This value is close to the experimental noise because it corresponds to the standard deviation of the residual between experimental values and best fits of the polymerase convoy model. For each simulated convoy, we fitted the time trace using the same analysis pipeline as for the experimental data, thereby calculating mean values and standard deviation for parameters t_{space}, v_{el} and t_{proc}.

We first simulated uniform polymerase spacing with a mean value of 4s, using an elongation rate of 4kb/min and a 3'-end processing time of 100 seconds. As shown in the Supplementary Table I, the analysis accurately recovered these input values. Next, we compared convoys with uniform polymerase spacing to convoys with exponential spacing, and varied parameters of convoys. In all cases, the mean spacing was properly identified. This indicates that the model performs well and is able to estimate the correct mean parameter values for convoys having either uniform or exponential distribution of spacing time. We also simulated convoys with variable number of polymerases (not shown). We found no correlation between polymerase spacing and the number of polymerases whithin convoys, as observed with the real data (Figure 2G). This is thus a property of the biological system and not an artefact of the analysis pipeline.

Supplementary Note 4: Mechanical modelling of DNA torsional forces during progression of a polymerase convoy

Main results: **(1)** in a convoy of active polymerases, any local desynchronization (pausing or slowing down of one polymerase locally affecting the spacing to neighboring polymerases) produces DNA supercoiling and a local increase of torsional energy, which in turn generates an apparent force sufficiently strong to restore the initial distance between the polymerases. **(2)** The ensuing collective behavior of the convoy enhances the stall force (that is, the hindering force capable of stopping polymerase activity) of polymerases in the convoy. Note that these results are valid whether polymerases are evenly spaced or not, but that the model does not explain how spacing is set.

Quantitative statements: **(1)** if the polymerase i (denoted $RNAP_i$ hereafter) is lagging and its distance to the following polymerase $RNAP_{i+1}$ decreases, supercoiling $\sigma(i,i+1)$ appears in the DNA segment separating them. The increase in torsional energy generates an effective force on $RNAP_i$ which is equal to $F(i+1 \rightarrow i) = 4\pi^2 k_B T\, l_{pt}\, \sigma(i,i+1)/h^2$ where k_B is the Boltzmann constant, T the temperature, l_{pt} the twist persistence length of DNA at this temperature, and h the pitch of the (relaxed) DNA double helix (that is, the distance $h = 3.4\,nm$ along the DNA axis corresponding to one turn, i.e. to $10.6\,bp$). If the distance between the polymerases $RNAP_i$ and $RNAP_{i+1}$ decreases by 1%, $\sigma(i,i+1) = 0.01$ and the force exerted on $RNAP_i$ is $F(i+1 \rightarrow i) \approx 13\,pN$. **(2)** Denoting K the stall torque hindering the activity of an isolated polymerase (related to the stall force F_S according to $K = F_S(h/2\pi)$), the stall torque of the polymerase within a convoy increases linearly with the convoy length, e.g. it equals $3K$ for the central polymerase of a convoy of 3 polymerases.

Detailed calculations (1): Effective forces generated by torsional coupling.

We consider a convoy of N elongating polymerases. Convoys are observed on actively transcribed genes, and presumably, chromatin in such regions is decondensed; no end constraints are considered. Distances are measured along the DNA axis. Polymerases enter DNA in $x = 0$ (TSs) and leave DNA in $x = L$ (termination site). The initial distances between successive polymerases are fixed by the promoter activity (or anything controlling the loading of polymerases onto DNA at the TSs). We label polymerases in the order of their loading onto DNA. We assume that polymerases actively perform a translation along the DNA axis, due to a strong viscous force preventing their rotation, while DNA screws into the polymerases. The viscous force against DNA rotation is assumed to be negligible. The viscous force preventing the rotation of a polymerase around DNA is possibly enhanced by the binding of other factors onto the polymerase. Accordingly, the linking number $Lk(i,i+1)$ of the DNA stretch separating the polymerases $RNAP_i$ and $RNAP_{i+1}$ is constant (at least in the absence of any topoisomerase activity). Its value is fixed by the distance $\Delta^0(i,i+1)$ separating $RNAP_i$ and $RNAP_{i+1}$ right after the loading of $RNAP_{i+1}$ according to $Lk(i,i+1) = \Delta^0(i,i+1)/h$ where h is the DNA pitch. When $RNAP_i$ and $RNAP_{i+1}$ move, any change in their relative distance $\Delta(i,i+1)$ reflects in a supercoiling of the DNA stretch separating them, equal to

$$\sigma(i,i+1) = \frac{hLk}{\Delta(i,i+1)} - 1 = \frac{\Delta^0(i,i+1)}{\Delta(i,i+1)} - 1 \qquad (1)$$

$\sigma(i,i+1)$ is simply the relative variation in the distance separating $RNAP_i$ and $RNAP_{i+1}$. We denote x_i (resp. x_{i+1}) the position of $RNAP_i$ (resp. $RNAP_{i+1}$) so that $\Delta(i,i+1) = x_i - x_{i+1}$ (see Figure 6E). The potential energy of torsion stored in this DNA stretch is thus

$$E_p(i,i+1) = \frac{1}{2} k_B T\, l_{pt} \left[\frac{2\pi}{h} \sigma(i,i+1) \right]^2 (x_i - x_{I+1}) \tag{2}$$

Plugging into the expression of $\sigma(i,i+1)$ yields

$$E_p(i,i+1) = \frac{2\pi^2}{h^2} k_B T\, l_{pt} \left[\frac{h^2 Lk^2}{x_i - x_{I+1}} - 2h\,Lk + (x_i - x_{I+1}) \right] \tag{3}$$

The force exerted on the ith polymerase by the $(i+1)^{th}$ one thus writes

$$F(i+1 \to i) = -\frac{\partial E_p(i,i+1)}{\partial x_i} = -\frac{2\pi^2}{h^2} k_B T\, l_{pt} \left[1 - \frac{h^2 Lk^2}{(x_i - x_{I+1})^2} \right] \tag{4}$$

Now $h^2 Lk^2 / (x_i - x_{i+1})^2 = \left[1 + \sigma(i,i+1)\right]^2$ and, because $\sigma(i,i+1) << 1$, we may approximate $\left[1 + \sigma(i,i+1)\right]^2$ by $1 + 2\sigma(i,i+1)$ so that

$$F(i+1 \to i) = 4\pi^2 k_B T \frac{l_{pt}}{h^2} \sigma(i,i+1) \tag{5}$$

Numerically, the prefactor of $\sigma(i,i+1)$ equals about $1300\,pN$ (with $h = 3.4\,nm$, $T = 300\,K$ and $k_B = 1.38\,10^{-23}\,JK^{-1}$). Hence for a relative decrease of 1% of the distance separating the two polymerases $RNAP_i$ and $RNAP_{i+1}$ (i.e. for $\sigma(i,i+1) = 0.01$), the force $F(i+1 \to i)$ pushing $RNAP_i$ forwards is already $13\,pN$. This is large since the maximal force that can be exerted by a molecular motor, e.g. a polymerase, is around $25-30\,pN$. In addition, during in vitro experiments, 17pN is the threshold above which yeast RNA polymerase stops when encountering an opposing force, and assisting or repelling forces of lower values accelerate or slow down RNA polymerases[3]. Since the propagation of DNA torsional stress is much faster than polymerase movement[4], the torsional forces and **the push-pull mechanism can thus strongly regulate the distance between successive polymerases.** Moreover, as the typical distance between two neighboring $RNAPs$ is about 280 bp (mean time spacing about 4s and mean velocity about 50 bp/s), a relative variation of 1% of their distance amounts to as few as 3 bp. This means that the distance between neighboring $RNAPs$ is exquisitely regulated by supercoiling constraints.

Detailed calculations (2): Collective effects and convoy propagation as a whole.

What remains to be solved now is the complete problem of transport of N harmonically coupled polymerases (the effective force is analogous to that exerted by a spring since the supercoiling $\sigma(i,i+1)$ equals the relative increase of the initial distance). This problem is reminiscent of some issues in traffic theory, e.g. the motion of an array of harmonically coupled vehicles (Wagner, 2001).

We first notice that the force F_i exerted on $RNAP_i$ is equivalent to a torque $\Gamma_i = F_i\left(\frac{h}{2\pi}\right)$.

Actually the force F_i and the torque $\Gamma_i = F_i\left(\frac{h}{2\pi}\right)$ form a “screw” (more precisely a “wrench”).

We denote K the torque exerted by an active polymerase. The work produced during the polymerization of one base pair is thus $W_{bp} = K\Delta\theta$ where $\Delta\theta \approx 35°$ is the angle between two consecutive base pairs in the DNA double helix. Denoting T_{bp} the duration of one cycle of polymerization, the angular velocity (with which DNA screws into the polymerase) writes $\omega = \Delta\theta / T_{bp}$. During this polymerization cycle, out-of-equilibrium chemical reactions, in particular NTP hydrolysis, drive a closed sequence of conformational transitions of the polymerase, producing an oriented motion and its transcriptional activity (corresponding to the progression of 1 bp along the DNA axis).

Denoting T_{turn} the duration needed for processing one complete turn of the double helix $T_{turn} = n_{turn}T_{bp}$ (where $n_{turn} = 10.6\,bp$ is the number of base pairs per DNA helix turn), it also comes $\omega = 2\pi / n_{turn}$. We assume that K is constant, only depending on the efficiency (energetic characteristics) of the chemical reactions. In particular K is independent of ω. Note that torques and forces can be measured by means of single-molecule micro-manipulations, while energies can be estimated from the characteristics of the chemical reactions.

The energy per turn $2\pi K$ is a constant that is fixed by the *number* of dNTP that is converted into PP_i (pyrophosphate) during one complete turn of the DNA into the polymerase (this number is 10.6 because one dNTP is converted into one PP_i for each transcribed base pair) while the angular velocity ω is strongly dependent on the dNTP *concentration* (Thomen et al., 2008).

Importantly, in this model the RNAP stall force is $F_S = K\left(\frac{2\pi}{h}\right)$. For yeast $RNAP$, $F_S = 17\,pN$, so that $K = 9\,pN.nm$.

Let us now consider a protein roadblock or any opposing action exerting a maximal resistive torque of magnitude M onto the polymerase $RNAP_i$. Let us also assume that $RNAP_i$ is in the middle of a large convoy of polymerases so that we can neglect end effects. And let us finally assume for simplicity that the initial distances between any two neighboring polymerases are all equal to one and the same value $\Delta°$.

(i) if $M < K$, $RNAP_i$ will keep on moving at the same velocity V through the roadblock.

(ii) if $M > K$, the roadblock will eventually block the polymerase activity and stop $RNAP_i$. At the same time both flanking polymerases $RNAP_{i-1}$ and $RNAP_{i+1}$ are still moving (at the same velocity V), so that $\Delta(i,i-1)$ and $\Delta(i,i+1)$ respectively increases and decreases with time. Hence $RNAP_{i-1}$ and $RNAP_{i+1}$ exerts respectively an increasing force $F(i-1 \to i)$ and $F(i+1 \to i)$ onto $RNAP_i$, both of the same magnitude F and both acting in the same direction: $RNAP_{i-1}$ pulling and $RNAP_{i+1}$ pushing $RNAP_i$. Therefore the resulting force acting on $RNAP_i$ is $2F$. At the same time each flanking polymerase also experiences an opposite force $-F$, namely $RNAP_{i-1}$ is pulled backwards and $RNAP_{i+1}$ is pushed backwards (see Figure 6E). The blocked polymerase $RNAP_i$ will resume its motion as soon as $K + 2F(h/2\pi) > M$, i.e. when the constraints have accumulated enough so that F reaches a threshold value

$F^* = (M-K)(\pi/h)$. Obviously F^* must be smaller than F_S F_S, which amounts to $M < 3K$. Otherwise both flanking polymerases would also stop before $RNAP_i$ has resumed its motion.

(iii) If $M > 3K$, both $RNAP_{i-1}$ and $RNAP_{i+1}$ stop before $RNAP_i$ has resumed its motion, so that the disturbance propagates and the remaining part of the convoy enters the scene: $RNAP_{i-2}$ starts pulling $RNAP_{i-1}$ while $RNAP_{i+2}$ starts pushing $RNAP_{i+1}$, both with the same force of increasing magnitude as $RNAP_{i-2}$ and $RNAP_{i+2}$ continue their motion. The same scenario as above applies and we deduce that the constraints keep on accumulating on $RNAP_i$ until either $RNAP_i$ resumes its motion (if $M < 5K$) or both $RNAP_{i-2}$ and $RNAP_{i+2}$ stop (if $M > 5K$).

(iv) We conclude that the RNAP stall torque within a convoy of N polymerases may be as large as NK.

The linear velocity V of the polymerase along the DNA axis is equal to $V = \omega(h/2\pi)$ where h is the local pitch of the DNA. The pitch h is hardly affected by supercoiling because, as shown before, relevant values of supercoiling during elongation are never larger than 1%. Therefore the pitch variations have a marginal effect on the velocity V as compared to the variations of ω due to pauses, roadblocks or opposing forces. Note that the pitch variations have also a marginal effect on the torque $\Gamma(i+1,i) = F(i+1,i)h/2\pi$ that results from the force $F(i+1 \to i)$.

It is worth noting that trafficking onto biological filaments is closely related to the motion of molecular motors. The basic model of out-of-equilibrium systems, ASEP, was introduced to

account for the relative motion of ribosomes onto mRNA[5]. Theoretical literature on polymerase motion simply applies this model to polymerases onto DNA. In particular only steric interactions are considered. However, a basic difference is that DNA experiences torsional constraints that do not affect the single-strand mRNA. While ASEP is a valid model for ribosome processivity, it is meaningless for polymerases. Accordingly, we do not expect to observe any synchronization and coherent motion for ribosomes (no ribosome convoys).

Supplementary Note 5: Cloning of the MS2 repeats and HIV-1 reporter plasmids

Two-variants of 16xMS2 were cloned, but one had two accidental mutations expected to inactivate binding to two of the stem-loops. The first repeat, MS2x16-XbaI, contained a unique XbaI site downstream of the repeat, while the second one, MS2x16-NheI, had an NheI site at the same position. Both repeats were further flanked by SpeI sites. The XbaI, NheI and SpeI sites have cohesive ends, and cloning of the SpeI fragment of MS2x16-XbaI into the NheI site of MS2x16-NheI thus generated a MS2x32 repeat containing a unique XbaI site downstream the repeats and flanked by SpeI sites, i.e. MS2x32-XbaI. Likewise, cloning of the SpeI fragment of MS2x16-NheI into the XbaI site of MS2x16-XbaI generated a MS2x32-NheI plasmid. Repetitions of these cloning steps then generated MS2x64 and MS2x128 repeats. Unlike the original MS2x24 sequence, these repeats were stable in bacteria and in cells.

HIV-1 plasmids were derived from pExo[6]. This plasmid contains an HIV-1 vector flanked by human genomic DNA. The vector contains two complete long terminal repeats (LTR) and is deleted for HIV-1 sequences between the viral packaging site and the RRE element. A cassette containing the FRT recombination sequence followed by a hygromycin resistance gene devoid of AUG initiator codon and followed by a polyA site was PCR amplified from pcDNA5 and cloned in the HIV-1 vector to generate the Flp-In plasmid pIntro. The original MS2x24 repeat of pExo was removed and a 45 bp linker containing five unique cloning sites was inserted in the NotI site of HIV-1 intron, between the packaging site and the RRE element. The MS2x128 repeat was then excised from pMK-MS2x128-XbaI with BamHI and EcoRV, and cloned in the HIV-1 intron of the modified pIntro, using the BamHI and SnaBI sites, to generate the plasmid pIntro-MS2x128.

Supplementary Note 6: Models containing branched or successive OFF states are kinetically indistinguishable

The following model describes the switches between different promoter states :

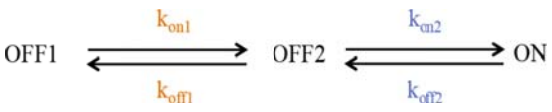

It is kinetically equivalent to the following model (2) :

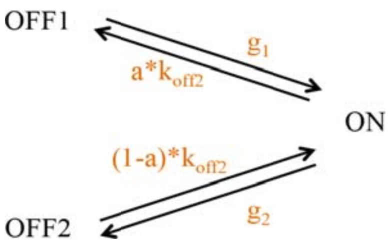

Indeed, the reaction scheme below has a bi-exponential solution for [ON]=f(t), when [OFF2] =1 and [OFF1]=[ON]=0 at t=0,

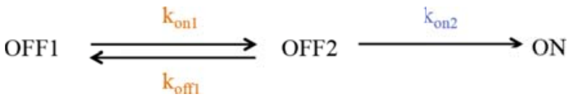

In addition, g1 and g2 are the roots of the polynomial x^2-S*x+P, with S = g1+g2 = $k_{on1}+k_{off1}+k_{on2}$, P = g1*g2 = $k_{on1}*k_{on2}$, and a = $k_{on2}*(k_{on1}$-g1)/(g1*(g2-g1)).

Supplementary References

1. Harper, F. & Puvion-Dutilleul, F. Non-nucleolar transcription complexes of rat liver as revealed by spreading isolated nuclei. *J Cell Sci.* **40**, 181-192 (1979).
2. McKnight, S. & Miller, O.J. Post-replicative nonribosomal transcription units in D. melanogaster embryos. *Cell* **17**, 551-563 (1979).
3. Zhou, J., Schweikhard, V. & Block, S. Single-molecule studies of RNAPII elongation. *Biochim Biophys Acta.* **1829**, 29-38 (2013).
4. Bécavin, C., Barbi, M., Victor, J. & Lesne, A. Transcription within condensed chromatin: Steric hindrance facilitates elongation. *Biophys J.* **98**, 824-833 (2010).
5. Ciandrini, L., Stansfield, I. & Romano, M. Role of the particle's stepping cycle in an asymmetric exclusion process: a model of mRNA translation. *Phys Rev E Stat Nonlin Soft Matter Phys.* **81**, 051904 (2010).
6. Boireau, S. *et al.* The transcriptional cycle of HIV-1 in real-time and live cells. *J Cell Biol.* **179**, 291-304 (2007).
